# What are the best clinical management strategies for cardiomyopathy? an umbrella review of systematic reviews and meta-analyses

**DOI:** 10.3389/fphar.2025.1544121

**Published:** 2025-07-25

**Authors:** Wanru Cheng, Jing Wang, Jie Sun

**Affiliations:** Department of Cardiology, The Second Affiliated Hospital, Zhejiang University School of Medicine, Hangzhou, Zhejiang, China

**Keywords:** cardiomyopathy, intervention, treatment, prognosis, umbrella review

## Abstract

**Background:**

The aim of this study is to summarize and evaluate the quality of evidence regarding the effectiveness and safety of different interventions for patients with cardiomyopathy, based on published meta-analyses through an umbrella review.

**Materials and Methods:**

The literature was searched via PubMed, Embase, Web of Science, and the Cochrane Library. Two reviewers evaluated the methodological quality of the included articles using the AMSTAR score. In addition, according to the Grading of Recommendations, Assessment, Development, and Evaluation (GRADE), evidence of each outcome was evaluated and graded as “high,” “moderate,” “low,” or “very low” quality for drawing conclusions. Additionally, each outcome was classified into four categories (classes I–IV and nonsignificant).

**Results:**

High-quality evidence suggested that for patients with cardiomyopathy, stem cell treatment could significantly improve left ventricular ejection fraction (LVEF), left ventricular ejection volume, 6-min walk distance (6-MWD), and New York Heart Association (NYHA) functional classification. High-quality evidence also suggested that for patients with dilated cardiomyopathy (DCM), adding traditional Chinese medicines (TCMs) such as Qili Qiangxin capsule (QQC), Shenmai injection (SMI), Zhigancao, and Shengmai to conventional Western medical treatment could significantly improve clinical effects, including LVEF, 6-MWD, and reductions in inflammatory indicators, left ventricular end-systolic diameter (LVESD), left ventricular end-diastolic diameter (LVEDD), and heart rate. In addition, high-quality evidence suggested that for patients with DCM, drugs such as atorvastatin, carvedilol, thyroid hormone, and L-carnitine could significantly improve LVEF and cardiac output and reduce C-reactive protein levels, systolic blood pressure, LVEDD, and left ventricular end-diastolic and end-systolic volumes. Furthermore, implantable cardioverter defibrillator (ICD) therapy could significantly reduce sudden cardiac death.

**Conclusion:**

High-quality evidence showed that cell therapy, atorvastatin, carvedilol, and thyroid hormone have significant improvement effects on the prognosis of cardiomyopathy. In addition, combining traditional Chinese medicines with conventional Western medicine therapy could significantly improve the effectiveness of conventional Western medicine therapy for cardiomyopathy.

## 1 Introduction

The European Society of Cardiology defines cardiomyopathy in their 2023 guidelines on cardiomyopathy management as a primary disorder of the heart muscle without established conditions like coronary artery disease, congenital heart disease, valvular heart disease, and hypertension (Arbelo et al., 2023). Cardiomyopathy frequently coexists with other heart conditions, including ischemic heart disease, valvular dysfunction, and hypertension. These conditions can coexist, indicating that having one does not rule out the possibility of developing another (Arbelo et al., 2023). Among the various types of cardiomyopathies, dilated cardiomyopathy (DCM) and hypertrophic cardiomyopathy (HCM) stand out as the most frequent cases. Many cardiomyopathies, such as cardiac amyloidosis, were considered rare in the past. With the improvement in the diagnostic level, the incidence rate is not uncommon as we recognized before. Although robust epidemiological data are lacking, DCM affects roughly 0.4% of the population, translating to approximately 1 in 250 individuals. HCM is slightly less common, with a prevalence of approximately 0.2% or roughly 1 in 500 people. Finally, arrhythmogenic right ventricular cardiomyopathy is the least frequent among these, affecting an estimated 0.04% of the population or approximately 1 in 2,500 individuals (McKenna and Judge, 2021). Cardiomyopathies encompass a diverse group of heart muscle disorders with various causes, complex clinical phenotypes, and multiple underlying mechanisms. There are many treatment methods to cardiomyopathy, including medications, instruments, and surgeries. DCM is the most common type of cardiomyopathy, and its causes can be quite diverse. Several factors can directly contribute to DCM, including autoimmunity, pathogenic or pathogenic gene variants, infections, exposure to toxins (such as cancer therapy, recreational drugs, and ethanol), tachyarrhythmias, and endocrinopathies. For patients diagnosed with a reduced left ventricular ejection fraction (LVEF) DCM, treatment guidelines recommend guideline-directed medical therapy. This therapy typically includes medications from four key classes: angiotensin receptor blockers (ARBs), mineralocorticoid receptor antagonists (MRAs), beta-blockers, and angiotensin-converting enzyme inhibitors (ACEIs), and in some cases, diuretic and sodium glucose cotransporter 2 inhibitor for improving prognosis. However, limited research exists on how effectively these therapies work in patient populations categorized by their specific genetic makeup (genotypes). Not all genotypes are suitable for these drug treatments. For example, some studies suggest that patients with DCM linked to LMNA gene mutations (LMNA-related DCM) may experience a lower response to conventional medical therapy. Therefore, personalized medicine for DCM has been proposed. Although exercise training can significantly enhance the functional capabilities and overall well-being of patients diagnosed with DCM, high-intensity exercise and participation in competitive sports may increase susceptibility to sudden cardiac death (SCD) in this population. Cardiac resynchronization therapy (CRT) and implantable cardioverter defibrillators (ICDs) are also indicated for individuals diagnosed with symptomatic DCM. It is a frequent justification for considering either heart transplantation or implantation of a durable left ventricular assist device as treatment options (Heymans et al., 2023). Recent studies have shown that adding traditional Chinese medicines (TCMs) can improve the prognosis of cardiomyopathy (Jin et al., 2024; Liu et al., 2023; Pi et al., 2017). For HCM, pharmacological therapy is basic treatment to improve a patient’s functional capacity and alleviate symptoms. For patients experiencing symptoms due to left ventricular outflow tract (LVOT) obstruction, drugs, surgery, and alcohol septal ablation (ASA) are used to improve symptoms (Ommen et al., 2024; Ommen et al., 2020). Other cardiomyopathies, such as Fabry disease, cardiac amyloidosis, inflammatory cardiomyopathy, and Chagas cardiomyopathy, usually require special treatments (Pieroni et al., 2021; Pieroni et al., 2024; Ruberg and Maurer, 2024; Bloom and Gorevic, 2023; Kittleson et al., 2020; Wechalekar et al., 2016; Tschöpe et al., 2021; Nunes et al., 2018; Ribeiro et al., 2012).

Despite the abundance of meta-analyses published in recent decades, including both observational studies and randomized controlled trials (RCT) investigating various treatments and outcomes for cardiomyopathy, several factors limit our ability to draw definitive conclusions. These limitations include shortcomings in study design, the heterogeneity of treatment approaches for different cardiomyopathy types, inconsistencies in findings across studies, and the lack of a universally accepted definition for cardiomyopathy. The therapeutic landscape for cardiomyopathy is rapidly evolving, with novel treatment modalities emerging for various sub-types. Although many types of cardiomyopathy have specific treatments, they may also share some common features. In this study, we aim to compare the outcomes of different intervention strategies and evaluate whether the addition of adjuvant or emerging therapies offers any real clinical benefit. Which intervention can improve the prognosis of cardiomyopathy better? This study used an umbrella review methodology to synthesize the findings from multiple meta-analyses on cardiomyopathy treatments to comprehensively assess the quality of existing evidence, potential biases within the studies, and the overall validity of the findings.

## 2 Methods

To ensure transparency and adherence to established methodological guidelines, the protocol for this umbrella review was prospectively registered with PROSPERO, registration number: CRD 42024541152.

### 2.1 Literature search

Our literature search was conducted in four databases: PubMed, Cochrane Library, Embase, and Web of Science. The search covered studies published from database inception through 31 October 2023. The detailed search strategy is provided in Figure 1 of the Supplement. Two reviewers independently searched for studies. We first screen for articles that might meet the requirements by reading the title and abstract, and then determine articles that meet the inclusion criteria by reading the entire text.

**FIGURE 1:**
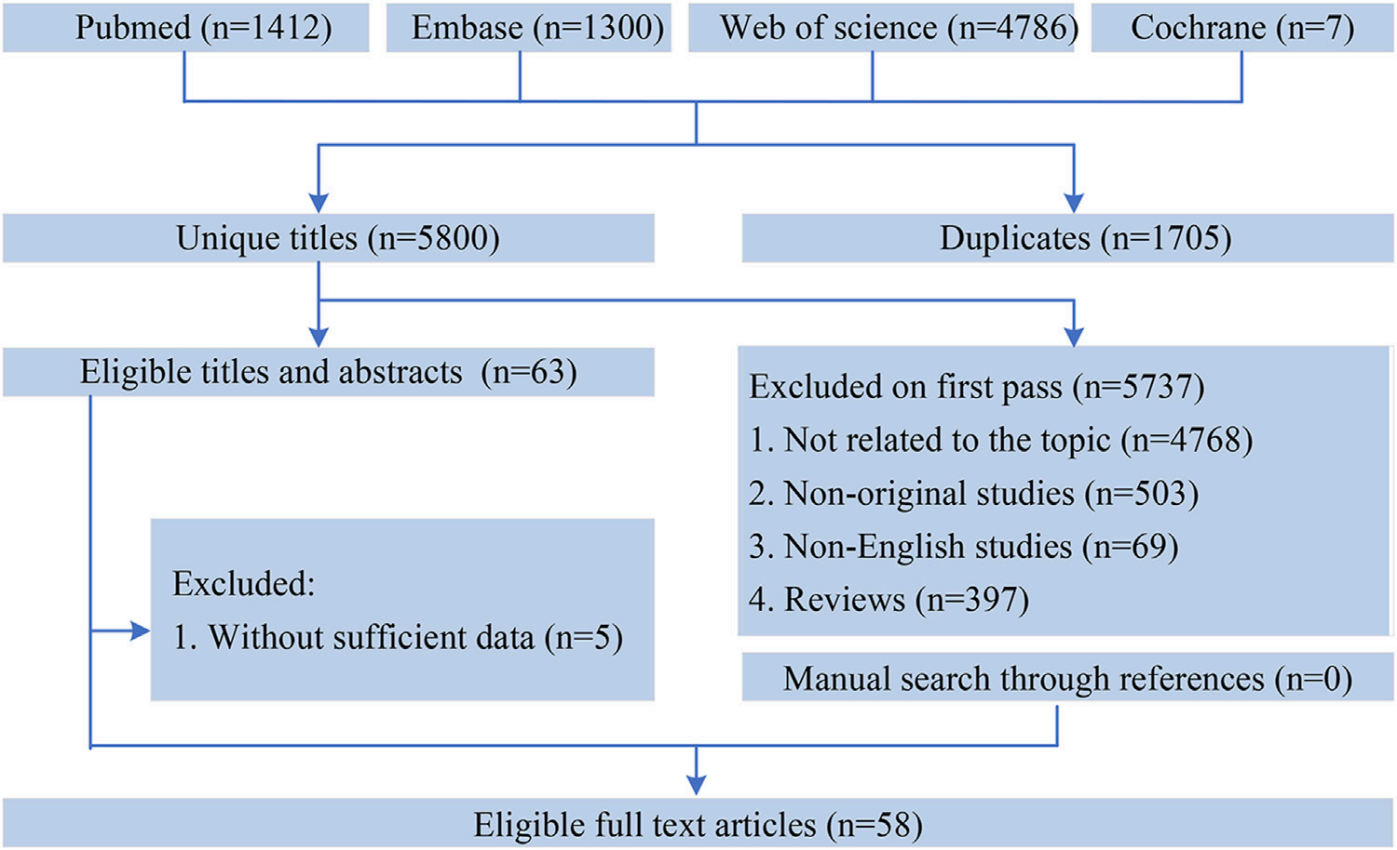
Diagram of literature-screening process.

### 2.2 Inclusion and exclusion criteria

We included the meta-analysis about the treatments of cardiomyopathy, which must have comparison of two or more treatment methods. The language was restricted to English. The exclusion criteria were as follows: nonhuman studies, studies with no comparison group, network meta-analyses, and non-original studies (letter, reviews, editorials, *etc*.).

### 2.3 Data extraction

To ensure data accuracy and minimize bias, two reviewers independently extracted the following information from each eligible study: author name, publication year, type of cardiomyopathy, intervention and control methods, treatment outcomes, number of studies included, participant numbers (intervention and control groups), study design distribution (case–control, cross-sectional, randomized controlled trial and cohort), and estimated summary effect measures [risk ratio (RR), odds ratio (OR), weighted mean difference (WMD), mean difference (MD), and standardized mean difference (SMD)], along with their corresponding 95% confidence intervals (CIs). We further evaluated the following aspects of each meta-analysis: the type of effect model used (random or fixed effects), the presence of heterogeneity among articles (using the I^2^ statistic and Q test p-value), and potential publication bias (evaluated through Egger’s test p-value or funnel plot analysis). For the same type of comparison, we chose the most recent article, and if the articles were all published within 2 years, we chose the one with the most references included. Any disagreement was determined by a third author.

### 2.4 Data analysis

We reanalyzed the WMD, SMD, MD, RR, or OR with 95% CI using random/fixed-effects models. Additionally, we assessed heterogeneity among studies by calculating the I^2^ statistic and Q test p-value. Furthermore, publication bias was evaluated using Egger’s regression test (studies with a sample size of 10 or more were included) to analyze potential small-study effects. This reanalysis focused on outcomes, number of studies, and participant numbers (control/intervention groups) reported in each meta-analysis. When reanalysis was not possible, we collected extracted data and evaluated both heterogeneity and publication bias to the best of our ability. We adopted a significance level of P < 0.10 for heterogeneity tests and P < 0.05 for all other statistical tests. We used Review Manager 5.4 for evidence synthesis and Stata 15.1 for Egger’s test and sensitivity analyses.

### 2.5 Quality assessment

AMSTAR, a well-established method for evaluating the methodological quality of systematic reviews and meta-analyses, was applied to evaluate the quality of methodology of the included articles by two reviewers (Shea et al., 2007). In addition, according to the Grading of Recommendations, our analysis used a four-category grading system to evaluate the quality of evidence for each outcome: class I (convincing evidence), class II (highly suggestive evidence), class III (suggestive evidence), class IV (weak evidence), and NS (nonsignificant). The specific criteria for classifying the evidence are outlined in Table 1 (Ioannidis, 2009; Huang et al., 2023). Moreover, we evaluated and graded the outcomes into “high,” “moderate,” “low,” or “very low” quality degrees, according to the Grading of Recommendations, Assessment, Development and Evaluation (GRADE) (Guyatt et al., 2011).

**TABLE 1:** Evidence classification criteria.

Evidence class	Description
Class I: convincing evidence	>1,000 cases (or >20,000 participants for continuous outcomes); statistical significance at P‹10^−6^ (random effects); no evidence of small-study effects and excess significance bias; 95% prediction interval excluded null value; no large heterogeneity (I^2^< 50%)
Class II: highly suggestive evidence	>1,000 cases (or >20,000 participants for continuous outcomes), statistical significance at P < 10^−6^ (random effects), and largest study with 95% confidence interval excluding null value
Class Ill: suggestive evidence	>1,000 cases (or >20,000 participants for continuous outcomes) and statistical significance at P < 0.001
Class IV: weak evidence	Remaining significant associations with P < 0.05
NS: nonsignificant	P > 0.05

## 3 Results

A total of 7,505 records were initially retrieved. After removing duplicates, 5,800 unique records remained and were screened independently by two reviewers based on titles, abstracts, and full texts. In the end, 58 studies met the inclusion criteria and were included in the final analysis (Figure 1). We extracted 159 corresponding outcomes, including 105 significantly associated outcomes and 54 nonsignificantly associated outcomes.

### 3.1 Traditional Chinese medicine

#### 3.1.1 High-quality evidence

##### 3.1.1.1 Qili Qiangxin capsule

The meta-analysis included 35 studies containing 3,334 patients with DCM, all published by J. Wei et al. in 2022. It demonstrated that compared with conventional Western medicine (CWM) [including ACEI/(angiotensin receptor inhibitors)/ARB, beta-blockers, diuretics, angiotensin receptor–neprilysin inhibitors, digoxin, and MRAs, and other treatments recommended by the guidelines] alone, the combination of conventional Western medicine and Qili Qiangxin capsule (QQC) (contains 11 crude herbs: *Astragalus mongholicus* Bunge, *Panax ginseng*, *Aconitum carmichaelii* Debeaux, *Salvia miltiorrhiza* Bunge, *Periploca sepium* Bunge, *Carthamus tinctorius*, *Citrus* × *aurantium*, *Neolitsea cassia*, *Polygonatum odoratum*, *Alisma plantago-aquatica*, and *Descurainia sophia*) could significantly improve 6-min walk distance (6-MWD), interleukin-6 (IL-6), tumor necrosis factor-α (TNF-α), and high-mobility group protein B1(HMGB1) (Wei et al., 2022).

Eleven trials including 828 patients reported the treatment effects on 6-MWD and 417/411 individuals with DCM in the intervention group and control group, respectively. We found that its heterogeneity was low (I^2^ = 0%; P = 0.81) and selected a fixed-effects model. The results showed that compared with CWM alone, QQC could significantly improve the MWD (MD: 41.93; 95% CI: 39.82–44.04; P < 10^–6^; AMSTAR 10; Evidence class IV; Figure 3). Moreover, they also exerted a subgroup analysis according to the treatment duration, and all selected the fixed-effects model (less than 12 weeks: I^2^ = 0%, P = 0.69; more than or equal to 12 weeks: I^2^ = 0%, P = 0.79). The intervention group had significant advantages in improving the 6-MWD than the control group either when the treatment duration was less than 12 weeks (MD = 42.04; 95% CI: 39.92 to 44.16; P < 0.00001) or more than or equal to 12 weeks (MD = 32.67; 95% CI: 13.49–51.85; P = 0.0008).

There were two trials on IL-6, with a total of 168 participants. The heterogeneity test showed I^2^ = 0% and p = 0.45, and the fixed-effects model was used for statistical analysis. The analysis proved that the intervention group was significantly better than the control group in reducing IL-6 in DCM patients (MD: −25.92; 95% CI: −31.35 to −20.50; P < 10^–6^; AMSTAR 10; Evidence class IV; Figure 3).

A total of 268 patients from four trials reported data on TNF-α. The fixed-effects model was used for statistical analysis (I^2^ = 45%; p = 0.14). The results showed that the experimental group could decrease TNF-α (MD: −5.04; 95% CI: −6.13 to −3.95; P < 10^–6^; AMSTAR 10; Evidence class IV) more significantly than the control group in patients with DCM (Figure 3).

Furthermore, three trials containing 178 patients reported HMGB1, and the fixed-effects model was used for analysis (I^2^ = 0%; p = 0.47). The result showed that the QQC group had an advantage in improving HMGB1 (MD: −4.34; 95% CI: −5.22 to −3.46; P < 10^–6^; AMSTAR 10; Evidence class IV) for patients with DCM (Figure 3) (Wei et al., 2022).

##### 3.1.1.2 Shenmai injection

The meta-analysis including 16 RCTs and 1,455 participants showed that compared with conventional treatment (beta-blockers, ACEI/ARB, angiotensin receptor–enkephalinase inhibitors, salt corticosteroid receptor antagonists, diuretics, digoxin, and other medications guided by the guidelines), combining Shenmai injections (SMI) (a modernized formulation of the TCM recipe Sheng-mai-san) with conventional treatment may lead to a more pronounced improvement in clinical outcomes and decrease left ventricular end-systolic diameter (LVESD) (Wang Y. et al., 2023).

**FIGURE 2:**
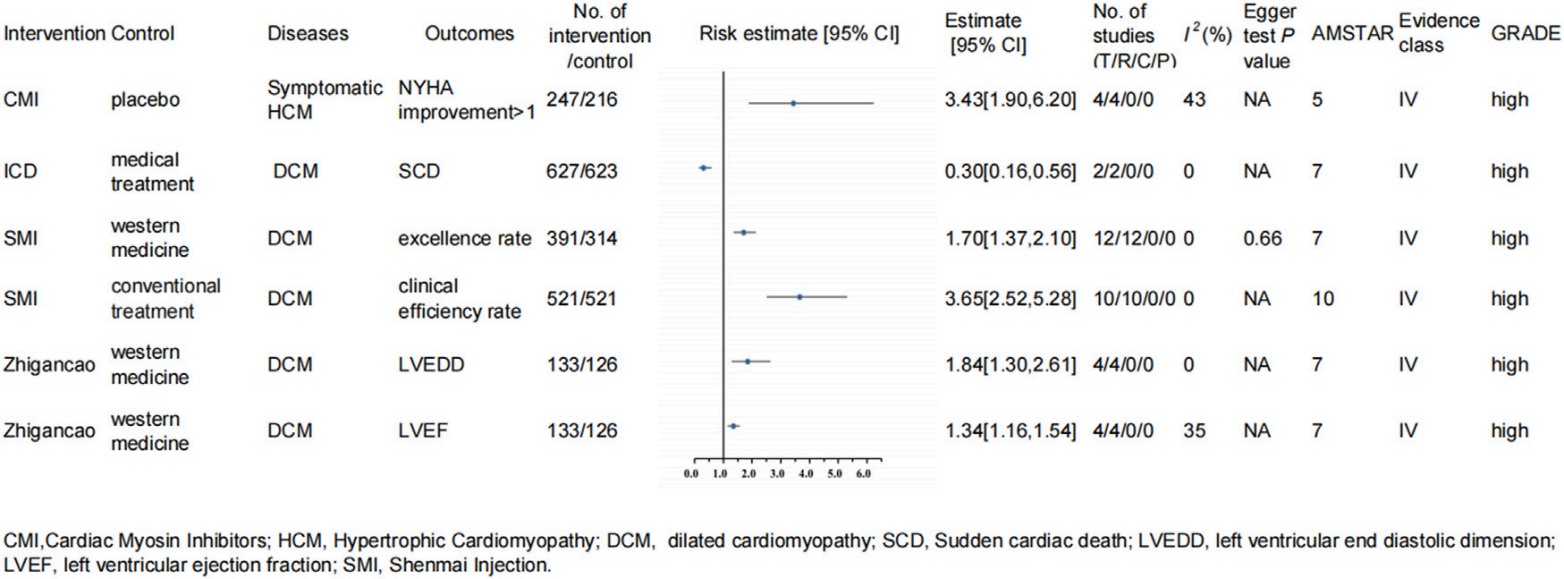
Forest plot of binary variable (high-quality evidence).

Ten RCTs involving 1,042 participants reported the clinical efficacy rate outcome involving 521 participants in the experimental group (SMI combined with conventional treatment) and 521 in the control group. The heterogeneity test showed I^2^ = 0% and P = 0.96; therefore, the fixed-effects model was used for the meta-analysis. The results illustrated that SMI combined with conventional treatment could improve the clinical efficacy rate of the DCM patients significantly (OR: 3.65; 95% CI: 2.52–5.28; P < 10^−6^; AMSTAR 10; Evidence class IV; Figure 2).

**FIGURE 3:**
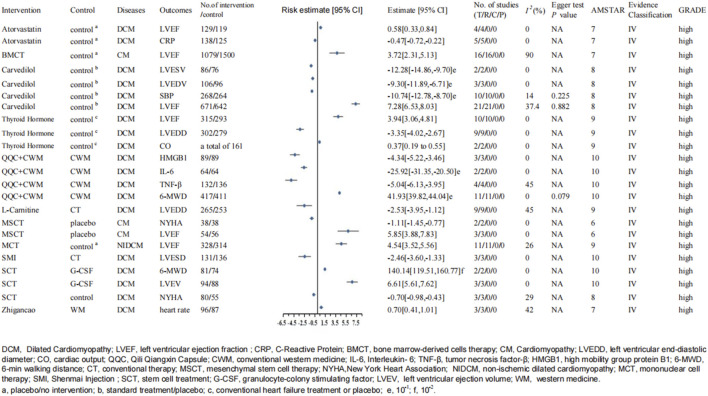
Forest plot of continuous variable (high-quality evidence).

A total of three RCTs reported on LVESD with 267 participants in total, including 131 and 136 participants in the intervention and control groups, respectively. The heterogeneity test showed I^2^ = 0% and P = 0.49, and the fixed-effects model was applied for meta-analysis. The meta-analysis demonstrated that conventional treatment combined with SMI treatment was more beneficial in reducing LVESD (MD: −2.46; 95% CI: −3.60 to −1.33; P = 0.0000215; AMSTAR 10; Evidence class IV; Figure 3) in patients with DCM than conventional treatment alone (Wang Y. et al., 2023).

##### 3.1.1.3 Shengmai

Twelve studies specifically investigated Shengmai decoction (one of the components of Yiqi Yangyin prescription), with 391 participants in the treatment group and 314 participants in the control group. The heterogeneity test showed I^2^ = 0% and P = 0.49, and the fixed-effects model was used for meta-analysis. Compared to conventional treatment (limiting salt, oxygen uptake, ACEI, diuretic, the foxglove/digoxin, the blood vessel dilation, and so on; different original studies might have a sight difference), conventional treatment combined with the Shengmai group had significant advantages in improving the excellence effect (total effective rate = excellence rate + effective rate) (RR: 1.70; 95% CI: 1.37, 2.10; P = 0.0000014; AMSTAR 7; Evidence class IV; Figure 2) (Zhou et al., 2017).

##### 3.1.1.4 Zhigancao

There were four studies focused on Zhigancao decoction, with 133 participants in the treatment group and 126 individuals in the control group. Adding Zhigancao decoction to conventional treatment could greatly improve LVEF (RR: 1.34; 95% CI: 1.16–1.54; P = 0.0001; AMSTAR 7; Evidence class IV; I^2^ = 35%; P-value for heterogeneity = 0.20) and decrease left ventricular end-diastolic diameter (LVEDD) (RR: 1.84; 95% CI: 1.30–2.61; P = 0.0006; AMSTAR 7; Evidence class IV; I^2^ = 0%; P-value for heterogeneity = 0.93) (Figure 2). They both chose the fixed-effects models (Zhou et al., 2017).

However, only three trials, containing 96 patients in the intervention group and 87 patients in the control group, reported the effect of Zhigancao on the heart rate. The fixed-effects model was used for statistical analysis (I^2^ = 42%; p = 0.18). The result indicated that adding Zhigancao to the conventional therapy could reduce the heart rate (MD: 0.70; 95% CI: 0.41–1.01; P = 0.00000301; AMSTAR 7; Evidence class IV; Figure 3) (Zhou et al., 2017).

#### 3.1.2 Moderate-evidence quality

##### 3.1.2.1 Qili Qiangxin capsule

**FIGURE 4:**
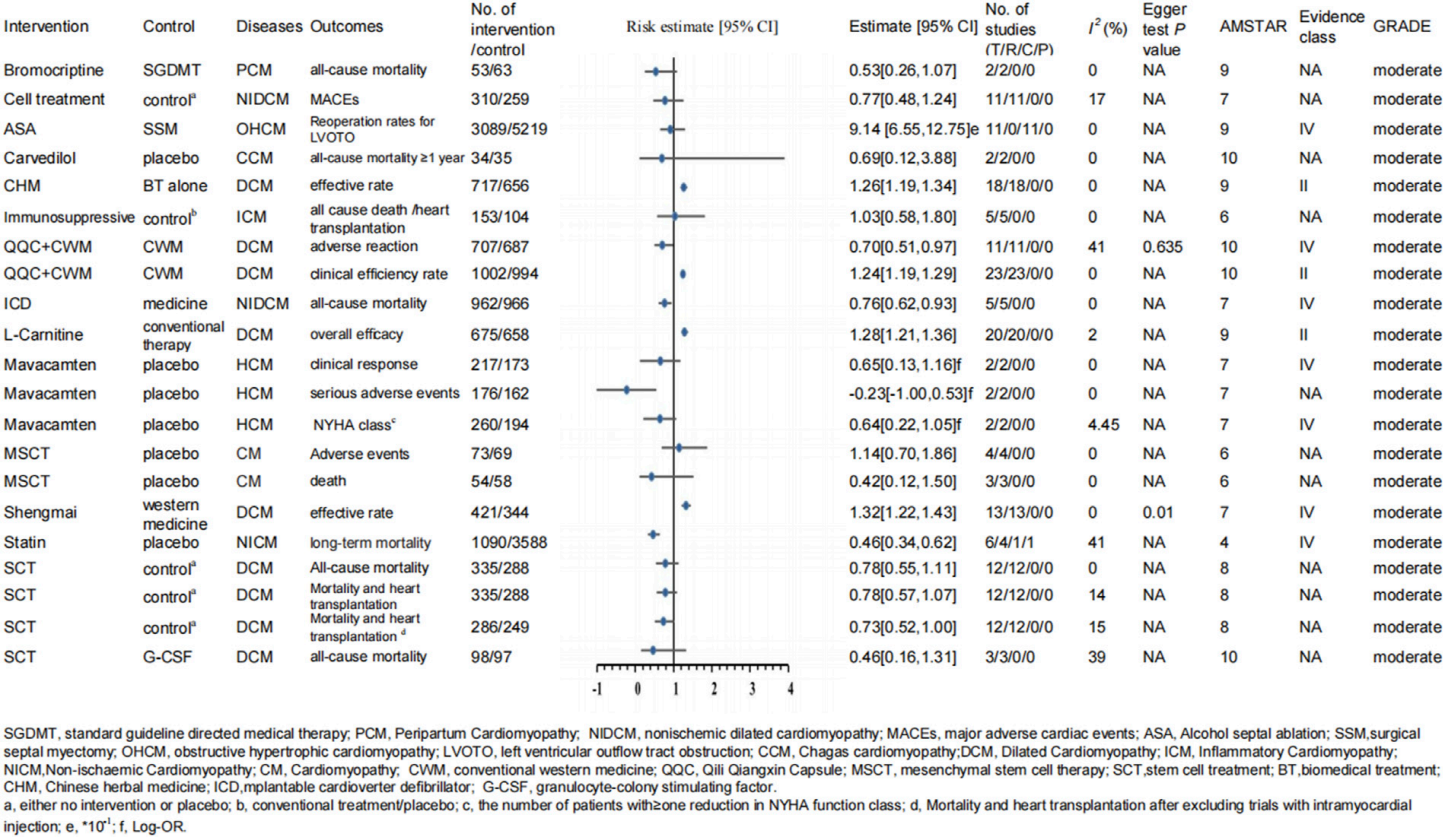
Forest plot of binary variable (moderate-quality evidence).

**FIGURE 5:**
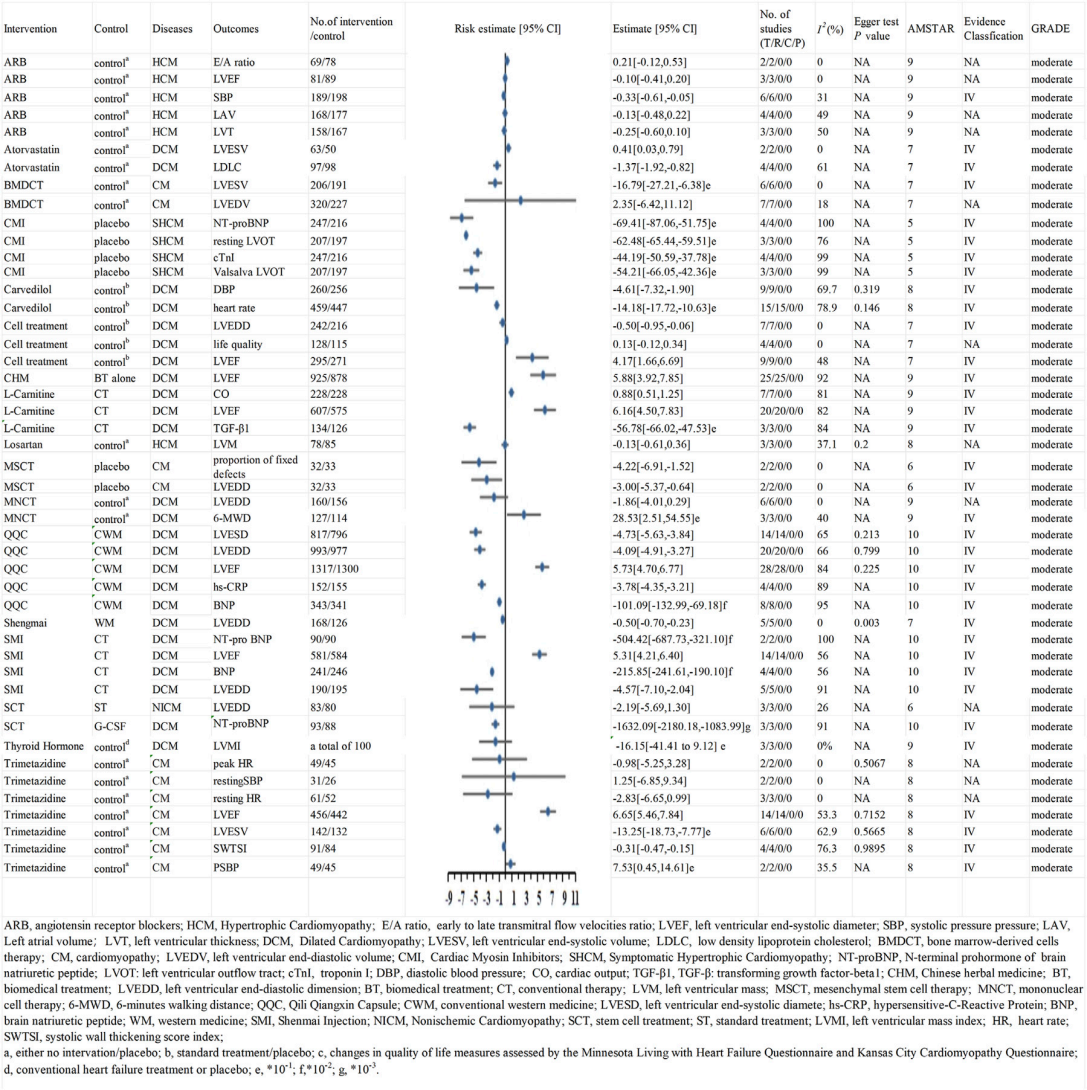
Forest plot of continuous variable (moderate-quality evidence).

The meta-analysis mentioned earlier revealed that compared with conventional Western medicine alone, the combination therapy (CWM and QQC) could markedly enhance the clinical efficiency rate (RR: 1.24; 95% CI: 1.19–1.29; P < 10^–6^) and LVEF levels (MD: 5.73; 95% CI: 4.70–6.77; P < 10^–6^) and reduce LVEDD (MD: −4.09; 95% CI: −4.91 to −3.27; P < 10^–6^), LVESD (MD: −4.73; 95% CI: −5.63 to −3.84; P < 10^–6^), plasma natriuretic peptide (BNP) (MD: −101.09; 95% CI: −132.99 to −69.18; P < 10^–6^), hypersensitive C-reactive protein (hs-CRP) levels (MD: −3.78; 95% CI: −4.35 to −3.21; P < 10^–6^), and adverse reactions (RR: 0.70; 95% CI: 0.51–0.97; P = 0.027) (Figures 4, 5) (Wei et al., 2022).

##### 3.1.2.2 Shenmai injection

Combining SMI with conventional treatment may lead to a significant decrease in LVEDD (MD: −4.57; 95% CI: −7.10 to −2.04; P = 0.0004), BNP (MD: −215.85; 95% CI: −241.61 to −190.10; P < 10^−6^)/N-terminal prohormone of brain natriuretic peptide (NT-proBNP) (MD: −504.42; 95% CI: −687.73 to −321.10; P < 10^−6^), and LVESD levels (MD: −2.46; 95% CI: −3.60 to −1.33; P = 0.0000215) and increase in LVEF levels (MD: 5.31; 95% CI: 4.2–6.40; P < 10^–6^) (Figure 5) (Wang Y. et al., 2023).

##### 3.1.2.3 Chinese herbal medicine

Compared with biomedical treatment (same as the previous text) alone, biomedical treatment plus Chinese herbal medicine (see Table 2) showed remarkable improvement in the effective rate (RR: 1.26; 95% CI: 1.19–1.34; P < 10^–6^) and LVEF (%) (MD: 5.88; 95% CI: 3.92–7.85; P = 0.0216) (Figures 4, 5) (Bai et al., 2013).

**TABLE 2:** Herbal ingredients of Chinese herbal medicine included in the meta-analysis.

Chinese herbal medicine	Herbal ingredient
Yiqifumaihuoxuelishuifang decoction	Ginseng, Hirudo, Radix glycyrrhizae preparata, Ramulus cinnamomi, Radix rehmanniae, Ophiopogon japonicas, Nardostachys chinensis Batal, Ligusticum chuanxiong Hort, Pepperweed seed, and Alisma orientale
Wenxin granule	Radix Codonopsis, Polygonatum kingianum, Panax Notoginseng Nardostachys chinensis Batal, and Succinum
Wenyangyiqihuoxuelishui decoction	Radix Aconiti Lateralis preparata, Atractylodes macrocephala, Angelica sinensis, Ramulus cinnamomi, Astragalus membranaceus, Poria cocos, Semen plantaginis, Radix glycyrrhizae preparata, Carthamus tinctorius and Ligusticum chuanxiong Hort
Huangqijianxin decoction	Astragalus membranaceus, Radix Aconiti Lateralis preparata, Radix Codonopsis, Atractylodes macrocephala, Poria cocos, Herba epimedii, Rhizoma zngiberis, Alisma orientale, Pepperweed seed, and Leonurus heterophyllus
Wenyanglishuihuoxuehuayu decoction	Radix Aconiti Lateralis preparata, Astragalus membranaceus, Ginseng, Ophiopogon japonicas, Ligusticum chuanxiong Hort, Atractylodes macrocephala, and Poria cocos
Xinyuan capsules	Polygonium multiflorum, Salvia miltiorrhiza, and Ophiopogon japonicus
Astragalus injection and oral liquid	Astragalus membranaceus
Qiliqiangxin capsules	Astragalus membranaceus, Ginseng, Radix Aconiti Lateralis preparata, Salvia miltiorrhiza, Pepperweed seed, Alisma orientale Polygonatum odoratum, Ramulus cinnamomi, Carthamus tinctorius, Cortex periplocae, and Perlcarpium citri reticulatae
Jianxin decoction	Radix Aconiti Lateralis preparata, Atractylodes macrocephala, Poria cocos, Radix paeoniae alba, Angelica sinensis, Semen persicae, Ramulus cinnamomi, Astragalus membranaceus, Ginseng, Radix glycyrrhizae preparata, Carthamus tinctorius, Rhizoma zingiberis recens, and Cinnamomum cassia
Baweitongluo granule	Hirudo, Pheretima, Ligusticum chuanxiong hort, Carthamus tinctorius, and Radix glycyrrhizae preparate
Yixinyin decoction	Astragalus membranaceus, Ramulus cinnamomi, Radix, Aconiti Lateralis preparata, Polyporus umbellatus, Poria cocos, Pepperweed seed, Ligusticum chuanxiong hort, Salvia miltiorrhiza, and Radix glycyrrhizae preparata
Fufanghuangqishengmaiyin decoction	Astragalus membranaceus, Radix Aconiti Lateralis preparata, Ophiopogon japonicas, Schisandra chinensis, Salvia miltiorrhiza, and Ginseng
Nuodikang capsules	Radix et rhizoma rhodiolae crenumatae
Astragalus injection and decoction	Astragalus membranaceus
Xinjihuoliyin decoction	Astragalus membranaceus, Radix Codonopsis, Ramulus cinnamomi, Ligusticum chuanxiong hort, Salvia miltiorrhiza Panax notoginseng, Coptis chinensis, and Forsythia suspense
Zhenwu decoction	Radix Aconiti Lateralis preparata, Pepperweed seed, Poria cocos, Atractylodes macrocephala, Radix paeoniae Alba, Salvia miltiorrhiza, Astragalus membranaceus, Fructus jujubae, and Rhizoma zingiberis Recens
She Xiang Bao Xin Wan	Moschus, Ginseng, Calculus bovis, Cinnamomum cassia, Styrax, Venenum Bufonis, and Borneolum syntheticum
Zhigancaojiaweifang decoction	Radix glycyrrhizae preparata, Rhizoma zingiberis Recens, Ramulus cinnamomi, Ginseng, Radix Rehmanniae, Colla corii asini, Ophiopogon japonicas, Fructus cannabis, Fructus jujubae, Astragalus membranaceus, Radix Aconiti Lateralis preparata, Poria cocos, Atractylodes macrocephala, and Salvia miltiorrhiza
Wenxin granule	Radix Codonopsis, Polygonatum kingianum, Panax notoginseng, Nardostachys chinensis Batal, and Succinum
Lingguishuganwuwei decoction	Astragalus membranaceus, Pueraria Lobata, Poria cocos, Ginseng, Ophiopogon japonicas, Ramulus cinnamomi, Lycopus lucidus, Salvia miltiorrhiza, Herba epimedii, Schisandra chinensis, Atractylodes macrocephala, Polygonatum odoratum, Cortex Periplocae, and Radix glycyrrhizae preparata
Astragalus injection and Shengmai injection	Astragalus membranaceus, Ginseng, Ophiopogon japonicas, and Schisandra chinensis
Zhigancaotangjiawei decoction	Ginseng, Ramulus cinnamomi, Ophiopogon japonicas, Radix rehmanniae, Colla corii asini, Fructus cannabis, Aucklandia lappa Radix polygalae, Schisandra chinensis, Rhizoma zingiberis recens, andFructus jujubae
Huangqishengmai decoction	Astragalus membranaceus, Ginseng, Cornus officinalis, Cortex periplocae, Ramulus cinnamomi, Poria cocos, Salvia miltiorrhiza, Polygonatum Kingianum, Polygonatum odoratum, and Radix glycyrrhizae preparata
Shenfu injection	Ginseng and Radix Aconiti Lateralis preparata
Huangqi granule	Astragalus membranaceus
Shuxuening injection and Ginkgo Biloba leaves Extract tablets	Folium ginkgo

##### 3.1.2.4 Shengmai

Compared with Western medicine alone, Western medicine combined with Shengmai could significantly reduce LVEDD levels (MD: −0.50; 95% CI: −0.70 to −0.23; P = 0.003) and increase the effective rate (RR: 1.32; 95% CI: 1.22–1.43; P = 0.01) for patients with DCM (Figures 4, 5) (Zhou et al., 2017).

#### 3.1.3 Low-evidence quality and very-low evidence

For patients with DCM, combining Shenmai injections (a modernized formulation of the TCM recipe Sheng-mai-san) with conventional treatment may increase 6-MWD (MD: 114.08; 95% CI: 42.32–185.85; P = 0.0019) (Wang Y. et al., 2023); Chinese herbal medicine (see Table 2) plus biomedical treatment (same as the previous text) showed remarkable decrease in LVEDD levels (MD: −2.78; 95% CI: −5.15 to −0.42; P < 10^–6^) compared with biomedical treatment alone (Bai et al., 2013); Shengmai combined with conventional treatment had advantages in improving LVEF levels (MD: 1.13; 95% CI: 0.55–1.70; P = 0.00013) but had no significant difference in the heart rate (MD: −0.54; 95% CI: −1.15 to 0.06; P = 0.0825) between the two groups for patients with DCM (Zhou et al., 2017).

### 3.2 Cell therapy

#### 3.2.1 High-evidence quality

##### 3.2.1.1 Stem cell treatment

The study published by R. Diaz-Navarro et al. in 2021 showed that compared with granulocyte–colony-stimulating factor (G-CSF) treatment, stem cell treatment (SCT) could significantly improve left ventricular ejection volume (MD: 6.61; 95% CI: 5.61–7.62; P < 10^−6^; AMSTAR 10; Evidence class IV) and 6-MWD (MD: 140.14; 95% CI: 119.51–160.77; P < 10^−6^; AMSTAR 10; Evidence class IV) (Figure 3). Three trials, containing 94 patients in the experimental group and 88 patients in the control group, reported the left ventricular ejection volume. The random-effects model was used for the analysis, and the heterogeneity test showed I^2^ = 0% and P = 0.54. At the same time, there were two RCTs reported about 6-WMD, with 81 and 74 participants in the intervention and control groups, respectively. The heterogeneity test showed I^2^ = 0% and P = 0.53, and the random-effects model was applied for analysis (Diaz-Navarro et al., 2021).

Another meta-analysis reported by L. Xia et al. in 2020 showed that SCT had an advantage in improving NYHA classification (WMD: −0.70; 95% CI: −0.98 to −0.43; P < 10^−6^; AMSTAR 8; Evidence class IV; Figure 3) compared with the control group. A total of three trials were included in the meta-analysis, with 80 and 55 patients in the intervention and control groups, respectively. The fixed-effects model was used for statistical analysis (I^2^ = 29%; P = 0.24) (Xia et al., 2020).

##### 3.2.1.2 Mesenchymal stem therapy

Mesenchymal stem cell therapy could remarkably increase LVEF levels (MD: 5.85; 95% CI: 3.88–7.83; P < 10^−6^; AMSTAR 6; Evidence class IV) and NYHA classification (MD: −1.11; 95% CI: −1.45 to −0.77; P < 10^−6^; AMSTAR 6; Evidence class IV) (Figure 3). The meta-analysis was published in 2019. Two RCTs reported LVEF, and three RCTs reported the NYHA classification. There were 54 and 46 patients with cardiomyopathy in the SCT and control groups, respectively, about LVEF. In addition, about NYHA classification, there were 38 patients in each group. Random-effects models were applied in both statistical analyses (I^2^ = 0% and P = 0.33 for NYHA; I^2^ = 0% and P = 0.84 for LVEF) (Li Y. et al., 2019).

##### 3.2.1.3 Mononuclear stem cell therapy

Furthermore, N. Nso et al. published a meta-analysis in 2022, which showed that compared with the control (placebo/NA) group (329 participants), patients with NICM treated with bone marrow mononuclear stem cell therapy (338 participants) showed a significant increase in LVEF (MD: 4.54%; 95% CI: 3.52–5.56; P < 10^−6^; AMSTAR 9; Evidence class IV; Figure 3). The heterogeneity showed I^2^ = 26% and p = 0.19 and selected the fixed-effects model (Nso et al., 2022).

##### 3.2.1.4 Cell therapy

The meta-analysis suggested that bone marrow-derived cell (BMC) therapy, compared with the control group, significantly improved LVEF (MD: 3.72%; 95% CI: 2.31–5.13; P < 10^−6^; AMSTAR 7; Evidence class IV; Figure 3). The heterogeneity test showed I^2^ = 90% and P < 0.00001, and the random-effects model was used for the meta-analysis. The types of cell therapy included bone marrow mononuclear cell (BMNC) and bone mesenchymal stem cell (BMSC). Furthermore, they reported the BMC efficacy according to different time durations. Subgroup analysis showed that the effect of BMC therapy on LVEF was observed at 1-month (MD: 3.57; 95% CI: 2.09 –5.05; P < 0.0001; I^2^ = 0%; P-value for heterogeneity = 0.84), 3-month (MD: 4.60; 95% CI: 3.27–5.94; P < 0.0001; I^2^ = 48%; P-value for heterogeneity = 0.05), 6-month (MD: 3.37; 95% CI: 0.27–6.46; P = 0.03; I^2^ = 89%; P-value for heterogeneity< 0.00001), and 12- to 60-month (MD: 3.59; 95% CI: 0.74–6.44; P = 0.01; I^2^ = 98%; P-value for heterogeneity< 0.00001) follow-up periods (Wang et al., 2019).

#### 3.2.2 Moderate-quality evidence

##### 3.2.2.1 Stem cell treatment

Another study showed that compared with G-CSF, SCT could significantly reduce BNP and NT-proBNP levels (MD: −1,632.09; 95% CI: −2,180.18 to −1,083.99; P < 10^−6^). Furthermore, the analysis did not detect any important difference in overall mortality rates (RR: 0.46; 95% CI: 0.16–1.31; P = 0.1495) between the SCT and G-CSF groups (Figures 4, 5) (Diaz-Navarro et al., 2021).

In addition, the meta-analysis including 12 RCTs with 623 patients indicated the effect of stem cell therapy compared with the control group. No striking differences were observed between the two groups in the reduction of LVEDD (WMD: 0.09 cm; 95% CI: 0.23–0.06; P = 0.207), all-cause mortality (RR: 0.78; 95% CI: 0.55–1.11; P = 0.16), mortality and heart transplantation (RR: 0.78; 95% CI: 0.57–1.07; P = 0.121), and mortality and heart transplantation after excluding trials with intramyocardial injection (RR: 0.73; 95% CI: 0.52–1.00; P = 0.069) (Figures 4, 5) (Xia et al., 2020).

Furthermore, another study indicated that compared with the standard treatment, the SCT group showed no significant decrease in LVEDD (MD: –2.19; 95% CI: –5.69 to 1.30; P = 0.2184; Figure 5) (Marqu et al., 2014).

##### 3.2.2.2 Mesenchymal stem cell therapy

As mentioned earlier, for patients with cardiomyopathy, mesenchymal stem cell therapy (MSCT) could remarkably reduce LVEDD (MD: –3.00; 95% CI: –5.37 to −0.64; P = 0.014) and the proportion of fixed defects (MD: –4.22; 95% CI: –6.91 to −1.52; P = 0.0026) (Figure 5). However, MSC treatment was not associated with a lower risk of death (RR: 0.42; 95% CI: 0.12–1.50; P = 0.169) or adverse events (RR: 1.14; 95% CI: 0.70–1.86; P = 0.598) compared with the placebo group (Figure 4) (Li Y. et al., 2019).

##### 3.2.2.3 Mononuclear stem cell therapy

Furthermore, Nso et al. published a meta-analysis in 2022, which showed that compared with the patients in the control (placebo/NA) group (329 participants), patients with NICM treated with bone marrow mononuclear stem cell therapy (338 participants) were able to walk 28.53 m more than the controls (MD: 28.53; 95% CI: 2.51–54.55; P = 0.03). However, there was no significant decrease in LVEDD (millimeter, mm) (MD: −1.86 mm; 95% CI: −4.01 to 0.29; P = 0.09) between the two groups (Figure 5) (Nso et al., 2022).

##### 3.2.2.4 Cell therapy

In addition, another meta-analysis included 11 RCTs with 574 subjects. In this article, the types of cell included the following: bone marrow mononuclear cells (BM-MNCs), bone marrow mesenchymal stem cells (BM-MSCs), and peripheral blood stem cells (PBSC). The analysis revealed that for patients with nonischemic dilated cardiomyopathy (NICM), compared to the control group, a significant increase in LVEF (MD: 4.17%; 95% CI: 1.66–6.69; P = 0.0012) and a decrease in LVEDD (SMD: −0.50; 95% CI: −0.95 to −0.06; P = 0.0295) were observed among patients receiving cell therapy (Figure 5). The analysis also revealed that there were no significant differences in patient-reported quality of life using either the Kansas City Cardiomyopathy Questionnaire (KCCQ) or the Minnesota Living with Heart Failure Questionnaire (SMD: 0.13; 95% CI: −0.12 to 0.34; P = 0.3067). Moreover, the investigation did not detect any substantial heterogeneity in the occurrence of major adverse cardiac events (MACEs) (OR: 0.77; 95% CI: 0.48–1.24; P = 0.2784) between the treatment and control groups (Figures 4, 5) (Tripathi et al., 2021).

Another meta-analysis suggested that bone marrow-derived cell therapy, compared with the control group, significantly decreased left ventricular end-systolic volume (LVESV) (MD: −16.79; 95% CI: −27.21 to −6.38; P = 0.0017), and there was no significant difference in LVEDV (MD: 2.35; 95% CI: −6.42 to 11.12; P = 0.5989) between the two groups of patients with cardiomyopathy (Figure 5) (Wang et al., 2019).

#### 3.2.3 Low-evidence and very-low-evidence quality

SCT may improve LVEF (WMD: 4.08%; 95% CI: 1.93–6.23; P = 0.0002) and 6-MWT (WMD: 101.49; 95% CI: 45.62–157.35; P = 0.0004) compared with the control group. However, no significant difference was observed between the two groups in the reduction of LVEDD (WMD: 0.09; 95% CI: 0.23–0.06; P = 0.207) and BNP levels (WMD: −326.66; 95% CI: −749.4 to 95.92; P = 0.128) for patients with DCM (Xia et al., 2020). Another study indicated that the SCT group also showed an important improvement in LVEF (MD: 4.87; 95% CI: 1.32–8.43; P = 0.0074) compared with the standard treatment group for patients with nonischemic cardiomyopathy (Marqu et al., 2014). In addition, another meta-analysis reported that for patients with NICM, cell therapy could ameliorate the functional capacity evaluated using 6-MWD (MD: 72.49 m; 95% CI: 3.44–141.53; P = 0.0028) (Tripathi et al., 2021).

### 3.3 Western medicine treatment

#### 3.3.1 High-evidence quality

##### 3.3.1.1 Atorvastatin

The analysis published in 2019 included five RCTs involving 138/125 participants with DCM, which revealed that compared with control groups (placebo/NA), atorvastatin treatment could reduce CRP levels (SMD: −0.47; 95% CI: −0.72 to −0.22; P = 0.0003; AMSTAR 7; Evidence class IV; Figure 3) significantly. The random-effects model was used for statistical analysis (I^2^ = 0%; P = 0.80). At the same time, the article also reported that atorvastatin treatment had significant advantages in improving LVEF (SMD: 0.58; 95% CI: 0.33–0.84; P = 0.000005; AMSTAR 7; Evidence class IV; Figure 3). There were four trials reported on LVEF and 129/119 patients with DCM in the intervention and control groups, respectively. The heterogeneity showed I^2^ = 0% and P = 0.52, and the random-effects model was applied to the analysis (Fu et al., 2020).

##### 3.3.1.2 Carvedilol

Another meta-analysis aimed to assess the clinical efficacy of carvedilol on DCM. A total of 15 studies specifically investigated the effects of carvedilol on LVEF, with 671 participants in the intervention group and 642 participants in the control group. The heterogeneity showed I^2^ = 37.4% and P = 0.044, and the fixed-effects model was applied to the analysis. The results showed that compared with the control groups (standard treatment/placebo), adding carvedilol showed significant improvement in LVEF (WMD: 7.28; 95% CI: 6.53–8.03; P < 10^–6^; AMSTAR 8; Evidence classification IV; Figure 3). There was no publication bias because of the P-value for the Egger test of 0.882 and that for the Begg test of 0.205.

The article also suggested that adding carvedilol had an advantage in lowering the systolic blood pressure (SBP) (WMD: −10.74; 95% CI: −12.78 to −8.70; P < 10^–6^; AMSTAR 8; Evidence classification IV; Figure3). Ten RCTs were involved to study the effect of carvedilol on SBP with 268 and 264 participants in the experimental and control groups, respectively. The heterogeneity showed P = 0.311 and I^2^ = 14.0%, and the fixed-effects model was used. The result of the Egger and Begg tests showed no publication bias (Egger P = 0.225; Begg P = 0.938).

In addition, a total of three studies provided analyzable data for LVEDV, including 109 and 96 patients in the intervention and control groups, respectively. The heterogeneity showed P = 0.601 and I^2^ = 0%, and the fixed-effects model was performed. The carvedilol group showed a significant decrease in LVEDV (WMD: −9.30; 95% CI: −11.89 to −6.71; P < 10^–6^; AMSTAR 8; Evidence classification IV) and LVESV (WMD: −12.28; 95% CI: −14.86 to −9.70; P < 10^–6^; AMSTAR 7; Evidence classification IV) (Figure 3). However, only two studies reported the LVESV, with 86 and 76 patients with DCM in the experimental and the control groups, respectively. The outcome was analyzed using the fixed-effects model because of the low heterogeneity (P = 0.597; I^2^ = 0%) (Li T. et al., 2019).

##### 3.3.1.3 Thyroid hormone

For patients with DCM, adding thyroid hormone therapy (triiodothyronine, thyroxine, or levothyroxine) carefully to standard heart failure medications (ACEI, beta-blockers, and diuretics) resulted in an important improvement in LVEF (WMD: 3.94; 95% CI: 3.06–4.81; P < 10^–6^; AMSTAR 9; Evidence classification IV) and CO (WMD: 0.37; 95% CI: 0.19–0.55; P < 10^–6^; AMSTAR 9; Evidence classification IV) (Figure 3) compared with conventional treatment alone. The intervention group was also associated with a considerable decrease in LVEDD (WMD: −3.35; 95% CI: −4.02 to −2.67; P < 10^–6^; AMSTAR 9; Evidence classification IV; Figure 3) (Chen et al., 2022).

##### 3.3.1.4 L-carnitine

Furthermore, another meta-analysis published in 2021 studied the effect of L-carnitine on patients with DCM, including nine RCTs with 265 and 253 patients in the intervention and control groups, respectively. The result showed that compared with conventional therapy, L-carnitine combined with conventional treatment could significantly reduce LVEDD (MD: −2:53; 95% CI: −3.95 to −1.12; P = 0.0005; AMSTAR 9; Evidence classification IV; Figure 3). The heterogeneity test showed I^2^ = 45% and P = 0.07, and the outcome was analyzed using the random-effects model (Weng et al., 2021).

##### 3.3.1.5 Cardiac myosin inhibitor

The research subjects of the last meta-analysis published in 2023 were the patients with symptomatic HCM, including four RCTs (one aficamten-focused trials and three mavacamten-focused) and 463 patients. The heterogeneity test showed I^2^ = 43% and P = 0.15, and the random-effects model was applied to the analysis. The result proved that the cardiac myosin inhibitor group showed an advantage in improving the proportion of patients, achieving NYHA class improvement ≥1 (OR: 3.43; 95% CI: 1.90–6.20; P = 0.00004; AMSTAR 5; Evidence classification IV; Figure 2) compared with the placebo group (Yassen et al., 2023).

#### 3.3.2 Moderate-evidence quality

##### 3.3.2.1 Atorvastatin

For patients with DCM, atorvastatin treatment showed significant advantages in reducing low-density lipoprotein cholesterol (SMD: −1.37; 95% CI: −1.92 to −0.82; P < 10^–6^) and could slightly increase LVESV (SMD: 0.41; 95% CI: 0.03–0.79; P = 0.0347) (Figure 5) compared with the control group (Fu et al., 2020).

##### 3.3.2.2 L-carnitine

Similarly, another article showed that adding L-carnitine may also be beneficial for patients with DCM. Compared with conventional therapy, L-carnitine combined with conventional treatment showed a significant increase in the cardiac output (CO) (MD: 0:88 L/min; 95% CI: 0.51–1.25; P = 0.00000295) and LVEF (MD: 6:16%; 95% CI: 4.50–7.83; P < 0.0001). Moreover, L-carnitine therapy could significantly decrease transforming growth factor-beta (TGF-β) (MD: −56:78 ng/L; 95% CI: −66.02 to −47.53; P < 10^–6^) (Figure 5) (Weng et al., 2021).

##### 3.3.2.3 Thyroid hormone

In addition, for patients with DCM, adding thyroid hormone did not appear to influence the left ventricular mass index (LVMI) (WMD: −16.15; 95% CI: −41.41 to 9.12; P > 0.05; AMSTAR 9; Evidence classification IV; Figure 5) and thyroid function compared with the control group (Chen et al., 2022).

##### 3.3.2.4 Carvedilol

Additionally, patients with DCM could also benefit from treatment with carvedilol. Compared with standard therapy/placebo treatment, adding carvedilol showed an advantage in reducing the heart rate (WMD: −14.18; 95% CI: −17.72 to −10.63; P < 10^–6^) and diastolic blood pressure (WMD: −4.61; 95% CI: −7.32 to −1.90; P = 0.0009) (Figure 5) (Li T. et al., 2019). However, for the patients with Chagas cardiomyopathy, carvedilol seemed to have no association with lower all-cause mortality with ≥1 year of follow-up (RR: 0.69; 95% CI: 0.12–3.88; P = 0.6776; Figure 4) compared with the placebo (Martí-Carvajal and Kwong, 2016).

##### 3.3.2.5 Angiotensin receptor blocker

For patients with HCM, the meta-analysis included data from seven RCTs, encompassing a total of 397 patients. Compared with the control group (placebo or standard non-ARB medication), the ARB treatment group (195 participants) could greatly reduce left ventricular mass (SMD: −0.71; 95% CI: −1.40 to −0.03; P = 0.044), left ventricular fibrosis (SMD: −0.60; 95% CI: −2.01 to 0.81; P = 0.40), and early diastolic velocity (SMD: −0.50; 95% CI: −1.70 to 0.70; P = 0.41) (Figure 5) (Abdelazeem et al., 2022). However, another meta-analysis suggested that losartan treatment showed no significant decrease in LVMI (SMD: −0.13; 95% CI: −0.61 to 0.36; P = 0.5935; Figure 5) compared with the control group among patients with HCM (Liu et al., 2022).

##### 3.3.2.6 Cardiac myosin inhibitor

The article reported that the effect of the cardiac myosin inhibitor group on patients with HCM showed a statistically significant difference in the baseline change in the mean LVOT gradient at rest (MD: −62.48; 95% CI: −65.44 to −59.51; P < 10^–6^) and the Valsalva LVOT gradient (MD: −54.21; 95% CI: −66.05 to −42.36; P < 10^–6^) between the cardiac myosin inhibitor group and the placebo group. The intervention group proved considerable reductions in mean percent change from baseline in NT-proBNP (MD: −69.41; 95% CI: −87.06 to −51.75; P < 10^–6^) and troponin I (MD: −44.19; 95% CI: −50.59 to −37.78; P < 10^–6^) (Figure 5) (Yassen et al., 2023).

Similarly, another meta-analysis suggested that the mavacamten group could significantly increase the clinical response (Log OR: 0.65; 95% CI: 0.13–1.16; P = 0.014) and the number of patients with a reduction of ≥1 NYHA function class (Log OR: 0.64; 95% CI: 0.22–1.05; P = 0.0028) and could not remarkably decrease the incidence rate of serious adverse events (Log OR: −0.23; 95% CI: −1.00 to 0.53; P = 0.5582) compared with the placebo group (Figure 4) (Rabiee Rad et al., 2023).

##### 3.3.2.7 Immunosuppressive therapy

For patients with inflammatory cardiomyopathy, the meta-analysis including five trials with a total of 316 patients showed that immunosuppressive therapy did not exhibit significant advantage in all-cause death or heart transplantation (OR: 1.03; 95% CI: 0.58–1.80; P = 0.9196; Figure 4) in the long term compared with the placebo and conventional therapy groups (Liu et al., 2005).

##### 3.3.2.8 Bromocriptine

For patients with peripartum cardiomyopathy, the additional use of bromocriptine to standard guideline-directed medical therapy (GDMT) appeared to have no significant decrease in all-cause mortality (RR: 0.53; 95% CI: 0.26–1.07; P = 0.0806; Figure 4) from two RCTs compared with GDMT alone (Kumar et al., 2023).

##### 3.3.2.9 Statin therapy

For patients with nonischemic cardiomyopathy, statin therapy was associated with significant higher late survival (HR: 0.45; 95% CI: 0.33–0.62; P < 10^−6^; I^2^ = 41%; P-value for heterogeneity = 0.13; Figure 4) compared with the placebo group (Deo et al., 2014).

##### 3.3.2.10 Trimetazidine

Beyond that, the meta-analysis published in 2018 aimed to study the effect of trimetazidine on cardiomyopathy. There was a statistical difference in LVEF, LVESV, systolic wall-thickening score index (SWTSI), LVESD, and LVEDD between the trimetazidine (TMZ, n = 456) group and the control (no TMZ/placebo, n = 442) group. TMZ treatment was superior in improving LVEF (MD: 6.65; 95% CI: 5.46–7.84; P < 10^−6^) and reducing LVESV (MD: –13.2552; 95% CI: −18.73 to −7.77; P = 0.0000019), SWTSI (MD: −0.3140; 95% CI: −0.47 to −0.15; P = 0.002), and peak SBP (MD: 7.5343; 95% CI: 0.4519–14.6167; P = 0.0373). There were no significant differences in the peak heart rate (MD: −0.9819; 95% CI: −5.2474 to 3.2836; P = 0.6483), resting heart rate (MD: −2.8305; 95% CI: −6.6556 to 0.9947; P = 0.1454), and resting SBP (MD: 1.2469; 95% CI: −6.8507 to 9.3446; P = 0.7589) (Figure 5) (Fan et al., 2018).

#### 3.3.3 Low- and very-low-evidence quality

##### 3.3.3.1 DCM

Atorvastatin treatment showed significant advantages in improving 6-MWD (SMD: 0.79; 95% CI: 0.27–1.31; P = 0.0031) and decreasing NT-pro BNP (SMD: −0.60; 95% CI: −1.18 to −0.01; P = 0.0427) but had no significant influence on LVEDV (SMD: 0.14; 95% CI: −0.37 to 0.64; P = 0.5887) (Fu et al., 2020). In addition, adding l-carnitine to conventional treatment could also reduce BNP (SMD: 1.71 ng/L; 95% CI: −3.02 to −0.40; P = 0.01) compared with conventional therapy (Weng et al., 2021). In addition, carvedilol could reduce LVEDD (WMD: −2.76; 95% CI: −4.89 to −0.62; P = 0.011) and LVESD (WMD: −3.63; 95% CI: −6.55 to −0.71; P = 0.015) significantly (Li T. et al., 2019).

##### 3.3.3.2 HCM

The ARB treatment group could greatly reduce SBP (SMD: −0.33; 95% CI: −0.61 to −0.05; P = 0.021), but LVEF (SMD: 0.10; 95% CI: −0.41 to 0.20; P = 0.53), left ventricular thickness (SMD: −0.25; 95% CI: −0.60, 0.10; P = 0.16), E/A ratio (SMD: 0.21; 95%CI: −0.12 to 0.53; P = 0.21), and left atrium volume (SMD: −0.13; 95% CI: −0.48 to 0.22; P = 0.47) did not display a statistically significant change compared with the control group (Abdelazeem et al., 2022). Another study indicated that mavacamten also had no significant influence on LVEF (SMD: −0.65; 95% CI: −1.50 to 0.20; P = 0.1323), peak oxygen uptake (SMD: 0.24; 95% CI: −0.35 to 0.82; P = 0.4241), and KCCQ (SMD: 0.43; 95% CI: −0.06 to 0.91; P = 0.0853) compared with the placebo group (Rabiee Rad et al., 2023). However, another study showed that patients in the symptomatic HCM cardiac myosin inhibitor group seemed to have reduced LVEF (MD: −6.31; 95% CI: −10.35 to −2.27; P = 0.023) evidently compared to those in the placebo group (Liu et al., 2022).

##### 3.3.3.3 Inflammatory cardiomyopathy

Immunosuppressive therapy might have a short-term (≤28 weeks) positive influence on LVEF improvement (WMD: 5.06%; 95% CI: −0.07% to 10.18%; P = 0.0532) but no significant benefit on long-term (>28 weeks) LVEF (WMD: 4.45; 95% CI: −5.25 to 14.15; P = 0.3667) and LVEDD either in the short-term (WMD: −0.87 mm; 95% CI: −8.29 to 6.55 in adult patients; P = 0.8174) or long-term (WMD: −0.52 mm; 95% CI: −3.64 to 2.60 in adult patients; P = 0.7427) influence compared with the control group (Liu et al., 2005).

Another article studied the effect of prednisolone and azathioprine (IPA) and showed that adding IPA to the optimal medical therapy (OMT) was not associated with better improvement of LVEF (MD: 9.9%; 95% CI: 1.8–21.7; P = 0.0973) and cardiovascular mortality (RR: 0.34; 95% CI: 0.08–1.51; P = 0.01439) compared with the OMT-alone group (Timmermans et al., 2020).

##### 3.3.3.4 Other cardiomyopathy

The meta-analysis including 552 participants with Fabry disease showed that there was no striking difference in LVMI improvement (SMD: −0.149; 95% CI: −0.431 to 0.132; P = 0.2998) between the enzyme replacement therapy (ERT, n = 267) and the control group (n = 285) (Lee et al., 2022).

The meta-analysis including 15 studies involving 2,765 patients with transthyretin amyloid cardiomyopathy suggested that the individuals who have undergone tafamidis treatment showed a significant positive influence on the lower risk of all-cause death or heart transplantation (RR: 0.44; 95% CI: 0.31–0.65; P = 0.00004) and composite endpoint (all-cause death, heart failure exacerbations, hospitalizations, heart transplant, and heart assist device implantation) (RR: 0.57; 95% CI: 0.42–0.77; P = 0.0003) compared to patients who have not undergone tafamidis treatment (Wang J. et al., 2023).

In addition, the meta-analysis suggested that the additional use of bromocriptine to standard GDMT appeared to considerably increase LVEF (MD: 12.56; 95% CI: 5.84–19.28; P = 0.0003) from two cohorts (MD: 14.25; 95% CI: 0.61–27.89; P = 0.0407) or two RCTs at follow-up and greater odds of left ventricular recovery (OR: 3.55; 95% CI: 1.39–9.10; P = 0.0081) but seemed to have no significant decrease in all-cause mortality (RR: 0.71; 95% CI: 0.30–1.67; P = 0.4359) from four cohort studies compared to the GDMT-alone group for patients with peripartum cardiomyopathy (Kumar et al., 2023).

The trimetazidine group showed an advantage in reducing LVEDD (MD: −0.4025; 95% CI: −0.55 to −0.26; P < 10^−6^) and LVESD (MD: −0.5828; 95% CI: −1.09 to −0.08; P = 0.0231) compared with the control group. However, there was no significant difference in LVEDV (MD: −5.2938; 95% CI: −13.8592 to 3.2717; P = 0.2248) between the two groups for patients with cardiomyopathy (Fan et al., 2018).

### 3.4 Invasive treatment and other treatment methods

#### 3.4.1 High-evidence quality

In 2023, D. A. Theuns et al. published the meta-analysis, which indicated that individuals with ICD use had a lower risk for SCD than those in the medical treatment group (HR: 0.30; 95% CI: 0.16–0.56; P = 0.0002; AMSTAR: 7; Evidence classification: IV; Figure 2). There were two RCTs, with 627 participants with NICM in the intervention group and 623 individuals in the control group. The heterogeneity test showed I^2^ = 0% and P = 0.48, and the random-effects model was applied for meta-analysis (Theuns et al., 2023).

#### 3.4.2 Moderate-evidence quality

##### 3.4.2.1 ICD

The same article as mentioned above also indicated that compared with the medical treatment group, individuals with ICD use could significantly reduce all-cause mortality (only RCT) (HR: 0.76; 95% CI: 0.62–0.93; P = 0.0082; Figure 4) (Theuns et al., 2023).

##### 3.4.2.2 Alcohol septal ablation versus surgical septal myectomy

For patients with HCM, alcohol septal ablation (ASA) was associated with less reoperation rates for LVOT obstruction (SMD: 9.14; 95% CI: 6.55–12.75; P < 10^–6^) than the surgical septal myectomy group (Figure 5) (Yokoyama et al., 2023).

#### 3.4.3 Low- and very-low-evidence quality

##### 3.4.3.1 ICD/CRT

However, if all trials were included in the study, not only the RCTs but also the result indicated that compared with the medical treatment group, ICD use for individuals with NICM could also significantly reduce all-cause mortality (HR: 0.72; 95% CI: 0.60–0.87; P = 0.0004). The study also reported that there was no statistically important difference between the CRT–defibrillator group and CRT–pacemaker in all-cause mortality (HR: 0.74; 95% CI: 0.47–1.16; P = 0.1934) and no significant difference between the CRT group and medical group in all-cause mortality (HR: 0.82; 95% CI: 0.67–1.00; P = 0.0542) (Theuns et al., 2023). For patients with pacing-induced cardiomyopathy or chronic right ventricular pacing, the upgrade-to-cardiac resynchronization therapy group showed significantly greater improvement in LVEF (SMD: 0.24; 95% CI: 0.05–0.43; P = 0.0133) than the de-novo CRT group. The analysis showed no statistically significant difference in response rates to cardiac resynchronization therapy (OR: 1.11; 95% CI: 0.79–1.56; P = 0.5475) between the two groups (Lu et al., 2022).

##### 3.4.3.2 Alcohol septal ablation versus surgical septal myectomy

There are 12 meta-analyses which studied the comparison between surgical septal myectomy (SM) and ASA for obstructive HCM patients. Among the 12 articles, we chose the latest and largest article. A total of 27 observational studies were included (15,968 patients). SM had a higher pacemaker implantation rate (HR: 1.68; 95% CI: 1.28–2.20; P = 0.0002) and lower long-term mortality with ≥5 years of follow-up (HR: 1.50; 95% CI: 1.04–2.15; P = 0.03). However, there were no significant differences in all-cause mortality with ≥1 year of follow-up (HR: 1.24; 95% CI: 0.88–1.76; P = 0.2189), 30-day mortality (HR: 0.99; 95% CI: 0.57–1.71; P = 0.9715), cardiovascular mortality (HR: 0.90; 95% CI: 0.49–1.64; P = 0.7341), rates of stroke (HR: 0.69; 95% CI: 0.28–1.71; P = 0.42), and rate of rehospitalizations due to heart failure (HR: 0.66; 95% CI: 0.21–2.05; P = 0.477) between the two groups (Yokoyama et al., 2023).

##### 3.4.3.3 Other methods

The meta-analysis focused on evaluating the effectiveness and safety of immunoadsorption therapy in patients with DCM. Compared with controls, the immunoadsorption treatment group could enhance LVEF (WMD: 6.01; 95% CI: 4.84–7.19; P < 10^–6^) and significantly reduce LVEDD (WMD: −3.62; 95% CI: −4.06 to −3.19; P < 10^–6^) and severity of symptoms according to the NYHA functional classification (SMD: −1.37; 95% CI: −1.73 to −1.02; P < 10^–6^). However, there was no significant effect on safety parameters (RR: 1.13; 95% CI: 0.58–2.19; P = 0.7195) (Bian et al., 2021).

In addition, another meta-analysis that included four RCTs involving 930 patients with DCM showed that compared with the standard therapy group, the cardiac contractility modulation device treatment group showed no significant influence on all-cause mortality (RR: 0.63; 95% CI: 0.29–1.35; P = 0.23) and that related to heart failure with reduced ejection fraction (RR: 0.65; 95% CI: 0.30–1.44; P = 0.2748) (Nadeem et al., 2020).

For patients with arrhythmogenic right ventricular cardiomyopathy, the meta-analysis did not detect a significant benefit of endo-epicardial ablation on acute procedural efficacy (OR: 2.74; 95% CI: 0.98–7.65; P = 0.054) or all-cause mortality (OR: 0.87; 95% CI: 0.09–8.31; P = 0.904) compared with the endocardial-only approach (Shen et al., 2020).

The research including four studies indicated that compared with the control group, the exercise-based training program had a significant higher exercise capacity (measured using peak VO2) (MD: 4.45; 95% CI: 3.50–5.39 mL/kg/min; P < 10^−6^) for patients with chronic Chagas cardiomyopathy (Calderon-Ramirez et al., 2023).

### 3.5 AMSTAR, GRADE, and Evidence class

To assess the methodological quality of the included studies, we used the AMSTAR scoring system. The median AMSTAR score of all outcomes was 8.08 (range 4–10; interquartile range 7–8) (Table 3). In addition, according to the GRADE rating criteria, 29 were rated as high-quality evidence (Figures 2, 3), 68 were rated as moderate-quality evidence (Figures 4, 5), 38 were rated as low-quality evidence, and 24 were rated as very-low-quality evidence. No separate figures were created for the low- and very-low-quality evidence due to their limited clinical relevance. In terms of evidence classification, for DCM, the effective rate (Chinese herbal medicine combined with biomedical treatment vs. biomedical treatment alone), overall efficacy (L-carnitine combined with conventional therapy vs. conventional therapy alone), and clinical efficiency rate (conventional Western medicine combined with Qili Qiangxin capsule vs. conventional Western medicine alone) were graded as class II. For the remaining 156 outcomes, 102 (65.4%) were identified as class IV and 54 (34.6%) were graded as nonsignificant. Because the participants of literature included were not enough, there was no class III. Moreover, the outcomes belonging to class II all had significant publication bias; therefore, we all assigned them to Evidence level II, and there was no level I.

**TABLE 3:** Assessments of AMSTAR scores.

Intervention	Comparison	Study	A priori design provided	Duplicate study selection and data extraction	At least two electronic databases searched	Status of publication used as an inclusion criterion	List of included and excluded studies provided	Characteristics of included studies provided	Scientific quality of included studies assessed	Scientific quality of the included studies used appropriately to form conclusions	Appropriate method to combine studies	Publication bias assessed	Conflict of interest included	Total AMSTAR score
ARB	Control (placebo/NA)	Abdelazeem et al. (2022)	1	1	1	0	1	1	1	0	1	1	1	9
ASA	SSM	Yokoyama et al. (2023)	1	1	1	0	1	1	1	0	1	1	1	9
Atorvastatin	Control (placebo/NA)	Fu et al. (2020)	0	1	1	0	1	1	1	0	1	1	0	7
BMCT	Control (NA)	Wang et al. (2019)	0	1	1	0	1	1	1	0	1	0	1	7
Bromocriptine	SGDMT	Kumar et al. (2023)	1	1	1	0	1	1	1	0	1	1	1	9
Cell treatment	Control (placebo/NA)	Tripathi et al. (2021)	0	1	1	0	1	1	1	0	1	0	1	7
IPA	OMT	Timmermans et al. (2020)	1	1	1	1	1	1	1	0	1	1	1	10
CCMT	ST	Nadeem et al. (2020)	0	1	1	0	1	1	1	0	1	1	1	9
CMI	placebo	Yassen et al. (2023)	0	1	1	0	1	0	0	0	1	0	1	5
Carvedilol	placebo	Martí-Carvajal and kwong (2016)	0	1	1	1	1	1	1	1	1	1	1	10
Carvedilol	ST/placebo	Li T. (2019)	0	1	1	0	1	1	1	0	1	1	1	8
CHM	BT alone	Bai et al. (2013)	0	1	1	0	1	1	1	0	1	1	1	9
QQC	CWM	Wei et al. (2022)	1	1	1	0	1	1	1	1	1	1	1	10
CRT	Medical treatment	Theuns et al. (2023)	0	1	1	0	0	1	1	0	1	1	1	7
CRT-D	CRT-P	Theuns et al. (2023)	0	1	1	0	0	1	1	0	1	1	1	7
Exercise	Control (NA)	Calderon-Ramirez et al. (2023)	1	1	1	1	1	1	1	0	1	1	1	10
Endo-epicardial ablation	Endocardial-only ablation	Shen et al. (2020)	0	1	1	0	1	1	1	0	1	1	1	8
ERT	NA	Lee et al. (2022)	0	1	1	0	1	1	0	0	1	0	1	6
ICD	Medicine	Theuns et al. (2023)	0	1	1	0	0	1	1	0	1	1	1	7
Immunoadsorption treatment	Control (NA)	Bian et al. (2021)	1	1	1	0	1	1	1	0	1	1	1	9
Immunosuppressive	Control (CT/placebo)	Liu et al. (2005)	0	1	1	0	0	1	1	0	1	0	1	6
Losartan	Control (placebo/NA)	Liu et al. (2022)	0	1	1	0	1	1	1	0	1	1	1	8
L-Carnitine	CT	Weng et al. (2021)	1	1	1	0	1	1	1	0	1	1	1	9
Mavacamten	Placebo	Rabiee Rad et al. (2023)	0	1	1	0	1	1	1	0	1	0	1	7
MSCT	Placebo	Li et al. (2019)	0	1	1	0	1	1	1	0	1	0	0	6
MNCT	Control (placebo/NA)	Nso et al. (2022)	0	1	1	0	1	1	1	1	1	0	1	8
SMI	CT	Wang et al. (2023)	1	1	1	0	1	1	1	1	1	1	1	10
Statin	Placebo	Deo et al. (2014)	0	1	0	0	0	1	1	0	1	0	0	4
Shengmai	WM	Zhou et al. (2017)	0	1	1	0	1	1	1	0	1	1	0	7
SCT	G-CSF	Diaz-Navarro et al. (2021)	0	1	1	1	1	1	1	1	1	1	1	10
SCt	ST	Marquis et al. (2014)	0	1	1	1	0	1	1	0	1	0	0	6
SCt	Control (NA)	Xia et al. (2020)	0	1	1	0	1	1	1	0	1	1	1	8
Trimetazidine	Control (NA)	Fan et al. (2018)	0	1	1	0	1	1	1	0	1	1	1	8
Tafamidis	Control (NA)	Wang et al. (2023)	1	1	1	0	1	1	1	0	1	1	1	9
Thyroid hormone	Control (CT/placebo)	Chen et al. (2022)	1	1	1	0	1	1	1	0	1	1	1	9
Upgrade CRT	de nove CRT	Lu et al. (2022)	0	1	1	0	1	1	1	0	1	1	1	8
Zhigancao	WM	Zhou et al. (2017)	0	1	1	0	1	1	1	0	1	1	0	7

### 3.6 Heterogeneity

A total of 47.7% (76) of all outcomes had significant heterogeneity (I^2^> 50% or P-value of Q test <0.1). Approximately 41 (25.8%) used the fixed-effects model and 118 (74.2%) used the random-effects model. Potential factors, including ethnicity, region, setting, age, sex, sample size, study design, study quality, duration of follow-up, and adjustment for confounding factors, could explain the heterogeneity of most outcomes.

### 3.7 Assessment of the risk of bias

We conducted Egger’s test for 16.4% (26) of all outcomes (whether there is a significant statistical difference or not) in our reanalysis. Among them, 21 had statistical differences, and only 4/21 had evidence of publication bias—Shengmai combined with Western medicine vs. Western medicine alone for patients with DCM: LVEF(P = 0.003), LVEDD (P = 0.003), effective rate (P = 0.01); trimetazidine vs. control for patients with cardiomyopathy: LVEDD (P = 0.0438). In addition, we reanalyzed the outcomes with AMSTAR ≥9 and included more than 10 articles. All outcome measures with more than 10 articles included in all continuous variables showed no significant publication bias. Moreover, some authors used funnel plots to detect whether there was publication bias.

## 4 Discussion

### 4.1 Findings of this umbrella meta-analysis

All meta-analyses about cell therapy showed that for patients with cardiomyopathy mainly DCM, this intervention could significantly improve 6-MWD, NYHA function classification, and LVEF, and reduce BNP/NT-pro BNP. However, all these articles proved that cell therapy could not significantly increase adverse events and death compared with the control group. There was no consistent result regarding LVEDV/LVEDD.

Another intervention was traditional Chinese medicine (such as Qili Qiangxin capsule, Shenmai injection, Shengmai preparations, and Chinese herbal medicine (see Table 2)). For patients with DCM, combining with some traditional Chinese medicine could significantly improve 6-MWD, LVEF, and clinical efficiency rate, and decrease BNP and inflammatory indicators (such as hs-CRP, IL-6, TGF-β, and HMGB-B1). In addition, this intervention did not increase the occurrence of adverse events. However, there was no consistent result regarding the heart rate.

Moreover, there was no doubt that some treatment methods targeting the etiology could significantly improve the prognosis of patients with cardiomyopathy. For example, for patients with HCM, cardiac myosin inhibitors (including mavacamten and aficamten) could significantly reduce LVOT pressure and NT-pro BNP and improve cardiac function (NYHA classification) but could not necessarily improve LVEF. At the same time, cardiac myosin inhibitors could not increase serious adverse events and improve life quality (assessed using the KCCQ score). Similarly, for patients with HCM, compared with surgical septal myectomy, alcohol septal ablation could sensibly decrease reoperation rates for LVOT obstruction, pacemaker implantation rate, and long-term mortality with ≥5 years of follow-up, but there was no difference in 30-day mortality, rates of stroke and cardiovascular mortality, rates of rehospitalizations due to heart failure, and all-cause mortality with ≥1 year of follow-up. Moreover, for transthyretin amyloid cardiomyopathy, tafamidis could significantly decrease all-cause death or heart transplantation and endpoint (all-cause death, hospitalizations, heart failure exacerbations, heart transplant, and heart assist device implantation). However, for patients with inflammatory cardiomyopathy, the combination of immunosuppressive treatment might have no significant influence on all-cause death or mortality, and at the same time, there was no significant improvement in LVEF (no matter long-term or short-term).

Otherwise, statin could significantly reduce long-term mortality for patients with nonischemic cardiomyopathy. Moreover, atorvastatin could significantly decrease low-density lipoprotein cholesterol and NT-pro BNP and improve 6-MWD. However, there was no significant difference in LVEDV. Furthermore, adding some adjunctive drugs such as carvedilol, thyroid hormone, trimetazidine, and L-carnitine, and exercise could improve the prognosis of patients with cardiomyopathy.

### 4.2 Cell therapy

Cell therapy is a new type of treatment for patients with cardiomyopathy, including ischemic cardiomyopathy, nonischemic cardiomyopathy, DCM, chronic Chagasic cardiomyopathy, diabetic cardiomyopathy, and cardiomyopathy caused by chemotherapy drugs. Substantial body of research, encompassing both animal models and human trials, supports the potential of cell therapy as a valuable approach to treat cardiomyopathy, but the results warrant further investigation. Despite the unclear mechanisms underlying cell therapy for cardiomyopathy, the primary therapeutic goal likely involves either (1) promoting the replacement of chronic myocardial scars with new muscle tissue (remuscularization) by transplanted cells or (2) stimulating the heart’s own repair mechanisms through paracrine signaling using these cells (Menasché, 2018). Not only animal investigations but also clinical studies supported the opinion that cell which in infancy can remove collagen and regenerate injured myocardium. Anti-fibrotic cytokine growth factors and matrix–metalloproteinases are the possible molecular mechanisms. Autologous cardiac, bone marrow-, and adipose tissue-derived stem cells have been demonstrated that these all can have significant positive impact on patients with cardiomyopathy.

Stem cell therapy, using either a patient’s own cells (autologous) or cells from a donor (allogeneic), has the potential to improve heart function by reducing scar tissue, promoting new blood vessel formation, and improving heart chamber shape through the release of beneficial factors (Vazir et al., 2019). Another review showed that mesenchymal stem cell therapy might be a prospective method for the prevention of diabetes-induced cardiomyopathy because mesenchymal stem cells have antiapoptotic, anti-fibrotic, and anti-inflammatory effects, as a promising strategy to improve heart function in patients with diabetes mellitus (da Silva et al., 2022). There were many RCTs which showed that cell therapy could significantly improve right ventricular function (Frljak et al., 2018), myocardial perfusion, LVEF, and 6-MWD, and sensibly decrease NT-pro BNP, mortality, or heart transplantation (Lezaic et al., 2015; Vrtovec et al., 2011; Vrtovec et al., 2013; Hamshere et al., 2015).

All of the above studies showed that cell therapy was expected to become an effective approach of treating cardiomyopathy from the root cause; however, all the studies indicated that cell therapy could remarkably improve cardiac function and reduce the adverse events at the same time. However, the number of people included in these analyses was not enough, so larger RCTs were required to definitely establish the safety and effectiveness of cell treatment. Moreover, all the meta-analyses included in our umbrella review were classified as evidence classification level IV. According to the GRADE rating criteria, 7 were rated as high-quality evidence, 18 as moderate-quality evidence, 5 as low-quality evidence, and 1 as very-low-quality evidence.

### 4.3 Chinese medicine

Traditional Chinese medicine displayed notable efficacy in combating cardiovascular diseases. The possible mechanisms of TCM in treating cardiomyopathy were as follows. Some traditional Chinese medicine could induce mitophagy, maintain mitochondrial homeostasis, and scavenge damaged mitochondria. TCM, including extracts, herbal preparations, and active monomers, may offer therapeutic potential for cardiovascular diseases by inducing mitophagy through various pharmacological mechanisms and signaling pathways (Wang et al., 2024).

Another article showed that Chinese medicines exerted cardioprotective effects by regulating the fatty acid metabolism. There was evidence suggesting that the disturbances of the cardiac fatty acid metabolism were important contributors in the development of cardiovascular diseases including cardiomyopathy. Dysfunctions in the cardiac fatty acid metabolism can lead to a cascade of detrimental effects, including inflammation, oxidative stress, energy deficit, and excessive apoptosis within the heart muscle. Modern research suggests that TCM interventions achieve their cardioprotective effects through the regulation of key protein expression in fatty acid metabolism pathways (Liu et al., 2023).

In addition, TCM had multicomponent and multitarget properties in different types of cells, ranging from HCM to diabetic heart disease. There was evidence showing that calcium, as we all know, acting as a second messenger, plays a critical role in the pathogenesis of cardiovascular diseases. Disruptions in calcium signaling within endothelial and vascular smooth muscle cells are a recognized contributor to the development of hypertension. The overload of calcium could induce arrhythmias, myocardial infarction, and apoptosis. Additionally, heart failure is associated with two critical abnormalities in calcium handling: enhanced sarcoplasmic reticulum calcium leakage and reduced calcium transient amplitude (Li et al., 2021).

As mentioned before, all relevant articles indicated that Qili Qiangxin capsule could improve cardiac function (mostly measured through echocardiogram) for patients with DCM. Although the meta-analysis included 35 RCTs, every original RCT included not more than 100 participants (Wei et al., 2022). Therefore, larger sample sizes and more rigorously designed RCTs are needed to confirm this result. Another study investigated the effects of Shenmai injections combined with conventional treatment for patients with DCM. This analysis implies that SMI may be beneficial for improving cardiac function; however, this analysis also had no enough samples, which included 16 RCTs; only two of them had a sample size exceeding 100, and most of them included not more than 50 samples in each group (Wang Y. et al., 2023). In addition, there are various types and complex components of TCM, and different people have different sensitivities. Although we observe that TCM has the possibility to improve the prognosis of DCM, further analysis is warranted to comprehensively evaluate potential adverse effects and explore inter-individual variability in response to this treatment.

### 4.4 L-carnitine

In the evidence classification II, L-carnitine was mentioned, which can keep a balance of cardiac metabolism by promoting mitochondrial β-oxidation and assist the transportation of long-chain fatty acids into the mitochondrial matrix. Moreover, L-carnitine exerts cardioprotective function by reducing inflammation, oxidative stress, and necrosis of cardiac myocytes. In addition, L-carnitine could also regulate intracellular enzyme release, endothelial integrity, calcium influx, and membrane phospholipid to maintain cellular homeostasis. There is a close relationship between cardiovascular disease and carnitine depletion, a metabolic and autosomal invisible hereditary disease. Therefore, carnitine is a promising strategy to improve cardiac arrhythmia, ventricular dysfunction, toxic myocardial injury, and ischemia–reperfusion injury (Wang et al., 2018). Another RCT investigated the effects of L-carnitine on patients with ischemic cardiomyopathy. The study population received a treatment of ACEI, diuretics, and digitalis previously. Individuals with ischemic cardiomyopathy obviously had higher red cell superoxide dismutase activity than healthy control patients. Moreover, red cell superoxide dismutase activity significantly increased in the group with L-carnitine, but no significant change was observed in the group without L-carnitine following 1 month. There was a considerable improvement in LVEF in both groups, but the group with L-carnitine had more significant increase after 1 month. L-carnitine could improve left ventricular systolic function and the erythrocyte superoxide dismutase activity for individuals with ischemic cardiomyopathy (Gürlek et al., 2000). An early study had found that L-carnitine could improve heart function in animal models (Whitmer, 1987). However, all original articles about L-carnitine included in the meta-analysis were of small sample sizes.

### 4.5 Alcohol septal ablation versus surgical septal myectomy

In addition, ASA versus surgical SM for obstructive HCM included the most meta-analyses, with a total of 12 articles, ranging from 2006 to 2023 (Yokoyama et al., 2023; Zeng et al., 2006; Alam et al., 2009; Agarwal et al., 2010; Leonardi et al., 2010; Liebregts et al., 2015; Singh et al., 2016; Poon et al., 2017; Osman et al., 2019; Bytyci et al., 2020; Jaiswal et al., 2022; Zheng et al., 2022). The following results were consistent: the ASA group had a higher rate of permanent pacemaker implantation (Alam et al., 2009; Poon et al., 2017; Osman et al., 2019; Bytyci et al., 2020; Jaiswal et al., 2022; Zheng et al., 2022) and re-intervention after surgery (Yokoyama et al., 2023; Poon et al., 2017; Bytyci et al., 2020; Jaiswal et al., 2022; Zheng et al., 2022). There was no difference in the long-term mortality rate (Agarwal et al., 2010; Liebregts et al., 2015; Bytyci et al., 2020), all-cause mortality rate (Yokoyama et al., 2023; Osman et al., 2019; Bytyci et al., 2020; Zheng et al., 2022), stroke (Bytyci et al., 2020; Jaiswal et al., 2022), and cardiovascular mortality rate (Osman et al., 2019; Bytyci et al., 2020; Jaiswal et al., 2022) between the two groups. However, there were no consistent conclusions in LVOT gradient reduction and NYHA function improvement. There were three meta-analyses (Zeng et al., 2006; Alam et al., 2009; Bytyci et al., 2020), indicting that SM could reduce LVOT pressure gradient more significantly, but the other three articles (Yokoyama et al., 2023; Agarwal et al., 2010; Zheng et al., 2022) held the opposite opinion, and another article (Poon et al., 2017) indicated that there was no difference between the two groups. In addition, two articles (Bytyci et al., 2020; Zheng et al., 2022) showed that SM was superior to the SM group in improving the cardiac function assessed using the grade of NYHA, but another article (Alam et al., 2009) suggested the opposite opinion, and one article (Zeng et al., 2006) showed no difference between the two groups. Moreover, some meta-analyses (Agarwal et al., 2010; Bytyci et al., 2020) showed that there was no difference in short-term mortality, but a meta-analysis (Jaiswal et al., 2022) showed that ASA had lower short-term mortality. Most meta-analyses (Osman et al., 2019; Bytyci et al., 2020) found that there was no significant difference in SCD between ASA and SM groups. However, one analysis (Leonardi et al., 2010) reported that after accounting for baseline characteristics, the odds of both all-cause death and SCD were lower in the ASA cohorts than in the SM cohorts. The meta-analysis (Yokoyama et al., 2023) published in 2023 showed that all-cause mortality with follow-up ≥ 5 years had favorable outcomes with SM; however, the result is only hypothesis generating given a subgroup analysis. Only one article (Zeng et al., 2006) published in 2006 studied that SM could reduce interventricular septal thickness more significantly.

### 4.6 Cardiac myosin inhibitor

HCM is a primary cardiomyopathy characterized by myocardial hypertrophy and impaired diastolic function directly because of abnormal sarcomeric function, which was caused by either encoding sarcomere protein gene mutations or other defects. Cardiac myosin is the fundamental motor protein for the function of the heart pump. Mavacamten, a small molecule, could inhibit the enzymatic activity of myosin, thus regulating cardiac function (Nag et al., 2023). Mavacamten acts as a selective allosteric inhibitor of cardiac myosin ATPase. By binding to a specific site on the myosin protein, it disrupts the formation of cross-bridges between actin and myosin filaments. This reduces myocardial contractility and improves overall myocardial energetics (Papadakis et al., 2020). Therefore, mavacamten may serve as a targeted drug for HCM.

An RCT called EXPLORER-HCM was conducted across 68 clinical cardiovascular centers in 13 countries. Patients (≥18 years old) were diagnosed with symptomatic obstructive HCM with an LVOT gradient ≥50 mmHg and NYHA functional class II–III. At 30 weeks, the mavacamten group showed a marked increase in the KCCQ overall summary (OS) score compared with the placebo group. Mavacamten treatment resulted in a significantly higher proportion of patients experiencing a very large improvement (KCCQ-OS ≥20 points) than placebo. In the mavacamten group, 36% (33 of 92) achieved this substantial improvement compared to only 15% (13 of 88) in the placebo group. After treatment was stopped, these gains returned to the baseline. Patients with symptomatic HCM treated with mavacamten experienced a substantial improvement in their overall health status compared to those receiving a placebo. Mavacamten emerged as a promising therapeutic approach, demonstrating significant improvement in patients’ clinical symptoms, physical function, social engagement, and overall quality of life (Olivotto et al., 2020). The EXPLORER-HCM trial also demonstrated that mavacamten could improve a range of cardiopulmonary exercise testing parameters beyond the carbon dioxide output, which indicated a lot of benefits on maximal exercise capacity (Wheeler et al., 2023). Another study suggested that for patients with obstructive HCM compared with the placebo group (n = 128), the mavacamten group (n = 123) could significantly improve diastolic function, including left atrial volume index (LAVI), lateral E/e', and systolic anterior motion. The reduction in LAVI was linked to the improvement of peak exercise oxygen consumption (Hegde et al., 2021).

### 4.7 Tafamidis

Transthyretin amyloid cardiomyopathy (ATTR-CM) is a well-established consequence of transthyretin protein misfolding and subsequent amyloid fibril deposition within the heart muscle (myocardium). This abnormal protein aggregation is the primary driver of disease pathology in ATTR-CM. Tafamidis, a protein stabilizer, could inhibit misfolding of the TTR protein and prevent tetramer dissociation and amyloidogenesis. Compared with the placebo group, tafamidis was associated with a significant reduction in mortality and hospitalizations, especially when used in the early stages (Ruberg and Maurer, 2024).

An analysis called ATTR-ACT RCT implied that compared with the placebo group, the tafamidis group had significant advantages in improving left ventricular stroke volume and reducing left ventricular global longitudinal strain, septal E/e', and lateral E/e'. In addition, compared with the placebo group, tafamidis (80 mg) could delay the progression of left ventricular systolic and diastolic functions over 30 months in individuals with ATTR-CM (Shah et al., 2024). Another multicenter RCT randomly assigned 441 individuals with transthyretin amyloid cardiomyopathy. Tafamidis treatment (n = 264) significantly reduced all-cause mortality and rates of cardiovascular-related hospitalizations compared to the placebo group (n = 177). Additionally, tafamidis could delay the decrease of 6-MWD and KCCQ-OS scores. However, the tafamidis and placebo groups had the similar incidence and types of adverse events (Maurer et al., 2018).

### 4.8 Carvedilol

More than 30 years ago, many studies reported on the treatment of heart disease with carvedilol, whether for animals or humans. Carvedilol could decrease oxidative stress and lower norepinephrine levels of coronary sinus selectively, thereby reducing cardiac adrenergic activity and improving endothelium-dependent vasodilation (Nakamura et al., 2002; Gilbert et al., 1996). These were possible mechanisms of carvedilol in treating patients with cardiovascular disease. At the same time, many RCTs reported that carvedilol could significantly improve left ventricular systolic function, left ventricular remodeling, left atrial function, and submaximal exercise tolerance, and reduce the incidence of ventricular arrhythmias (Metra et al., 1994; Cice et al., 2000; Kasama et al., 2007; Paraskevaidis et al., 2007).

## 5 Limitations

There were limitations in this umbrella review. First, the definition of cardiomyopathy is not uniform, and there are many types of cardiomyopathy. Moreover, different treatments for cardiomyopathy vary. We cannot analyze the treatment methods for all types of cardiomyopathy. The description in the article is also quite scattered and cannot provide a detailed comparison of a particular cardiomyopathy. Second, some analyses included less number of studies, and this may impact the accuracy of results. Third, we excluded systematic reviews based on network meta-analyses. Although network meta-analyses offer a valuable tool for comparing multiple interventions simultaneously, integrating their results with findings from conventional pairwise meta-analyses remains a developing area. Fourth, umbrella reviews are inherently limited by the number of outcomes that can be realistically assessed due to the broad scope of the research they encompass. Fifth, some meta-analysis studies included literature from several years ago, not from the recent years. Furthermore, most evidence levels are level IV, and part of the effect indicators’ AMSTAR scores did not exceed five points. Next, there are many treatment methods for cardiomyopathy, and our article only analyzes a small portion of them that have undergone meta-analysis research. Moreover, the articles about traditional Chinese medicine were all studied in China and included all Chinese people. Finally, some original articles did not have enough sample size, which may lead to inaccurate results. Therefore, the conclusions need to be drawn from a larger population.

## 6 Conclusion

High-quality evidences showed that for patients with DCM, atorvastatin could significantly improve LVEF and reduce CRP; carvedilol also could significantly improve LVEF and reduce SBP, LVEDV, and LVESV; at the same time, thyroid hormone could also significantly improve LVEF and cardiac output and reduce LVEDD; L-carnitine also could markedly reduce LVEDD; furthermore, ICD therapy could significantly reduce sudden cardiac death. Finally, an emerging drug called cardiac myosin inhibitor could significantly improve symptoms in patients with symptomatic HCM measured using NYHA classification.

In addition, high-quality evidence also suggested that for patients with DCM, adding the Qili Qiangxin capsule to conventional Western medicine therapy could significantly improve 6-WMD and reduce IL-6, TNF-α, and HMGB1; adding SMI to conventional treatment could lead to a pronounced improvement in clinical outcomes and decrease in LVESD; in addition, adding Zhigancao to Western treatment could obviously improve LVEF and reduce LVEDD and heart rate; meanwhile, adding Shengmai also had significant advantages in improving the excellence effect.

High-quality evidence also indicated that for patients with cardiomyopathy, bone marrow-derived cell therapy could significantly improve LVEF; MCSC therapy also could remarkably improve LVEF and NYHA functional classification; moreover, for patients with nonischemic cardiomyopathy, MNSC therapy could also significantly improve LVEF; finally, for patients with DCM, stem cell therapy could significantly improve left ventricular ejection volume, 6-MWD, and NYHA classification.

## Data Availability

The original contributions presented in the study are included in the article/Supplementary Material; further inquiries can be directed to the corresponding author.

## Glossary

6-MWD: 6-min walk distance
ACEI: angiotensin-converting enzyme inhibitor
AMSTAR: A MeaSurement Tool to Assess systematic Reviews
ARBs: angiotensin receptor blockers
ASA: alcohol septal ablation
ATTR-CM: transthyretin amyloid cardiomyopathy
BMC: bone marrow-derived cell therapy
BMNC: bone marrow mononuclear cell
BM-MNCs: bone marrow mononuclear cells
BNP: brain natriuretic peptide
CI: confidence interval
CO: cardiac output
CRP: C-reactive protein
CRT: cardiac resynchronization therapy
cTnI: troponin I
CWM: conventional Western medicine
DCM: dilated cardiomyopathy
ERT: enzyme replacement therapy
E/A ratio: early-to-late transmitral flow velocity ratio
G-CSF: granulocyte–colony stimulating factor
GDMT: standard guideline-directed medical therapy
GLS: global longitudinal strain
GRADE: Grading of Recommendations, Assessment, Development, and Evaluation
HCM: hypertrophic cardiomyopathy
HMGB1: high-mobility group protein B1
hs-CRP: hypersensitive C-reactive protein
ICD: implantable cardioverter defibrillator
IL-6: interleukin-6
IPA: prednisolone and azathioprine
KCCQ: Kansas City Cardiomyopathy Questionnaire
LVEDD: left ventricular end-diastolic dimension
LVEDV: left ventricular end-diastolic volume
LVEF: left ventricular ejection fraction
LVESD: left ventricular end-systolic diameter
LVESV: left ventricular end-systolic volume
LVMI: left ventricular mass index
LAVI: left atrial volume index
LVOT: left ventricular outflow tract
MACEs: major adverse cardiac events
MD: mean difference
MRAs: mineralocorticoid receptor antagonists
MSCT: mesenchymal stem cell therapy
NT-proBNP: N-terminal prohormone of brain natriuretic peptide
NYHA: New York Heart Association
NICM: nonischemic dilated cardiomyopathy
OMP: optimal medical therapy
OR: odds ratio
PBSC: peripheral blood stem cell
QQC: Qili Qiangxin capsule
RR: risk ratio
SCD: sudden cardiac death
SCT: stem cell treatment
SM: surgical septal myectomy
SMI: Shenmai injection
SMD: standard mean difference
SWTSI: systolic wall-thickening score index
TCM: traditional Chinese medicine
TGF-β: transforming growth factor-beta
TNF-α: tumor necrosis factor-α
WMD: weighted mean difference
